# Impact of the RaS-RiPP tryglysin and culturing conditions on ex-vivo oral microbiomes

**DOI:** 10.1038/s41522-025-00794-8

**Published:** 2025-08-04

**Authors:** Britta E. Rued, Achal Dhariwal, Brett C. Covington, Mohammad R. Seyedsayamdost, Sophie A. Krivograd, Russell P. Pesavento, Michael J. Federle, Fernanda C. Petersen

**Affiliations:** 1 Department of Veterinary Microbiology and Preventive Medicine, Iowa State University, Ames, IA USA; 2 Institute of Oral Biology, University of Oslo, Oslo, Norway; 3 Department of Chemistry, Princeton University, Princeton, NJ USA; 4 Department of Oral Biology, College of Dentistry, University of Illinois, Chicago, IL USA; 5 Department of Pharmaceutical Sciences, University of Illinois, Chicago, IL USA

**Keywords:** Microbiology, Dentistry

## Abstract

Natural products are ubiquitously produced by many species that we encounter during our daily lives. One genus, *Streptococcus*, can produce a wide array of quorum sensing linked natural products known as RaS-RiPPs (ribosomally synthesized and post-translationally modified peptides). Their production is triggered by the induction of an Rgg-SHP quorum sensing system, which senses the presence of SHPs (short hydrophobic peptides) and induces the gene expression of these operons. Previous work has found that streptococcal RaS-RiPPs modulate the growth of different streptococci and might play a role in antibiotic tolerance. This is of particular importance to the oral microbiome, where streptococci are a predominant genus. This study provides the first report on attempts to study the impact of the RaS-RiPP Tryglysin A on ex-vivo oral systems and explores important factors to consider when culturing these systems. We explore how medium selection, atmosphere, growth model, and saliva amount can impact the presence of both bacterial and fungal species. These studies provide the groundwork for determining how RaS-RiPP producing Streptococci might impact the composition and function of oral microbiome communities, as well as important aspects to consider when culturing ex-vivo oral systems.

## Introduction

The oral cavity is a site of colonization for many important microbes, both bacterial and fungal^1–4^, which can have huge impacts on human health. These impacts include the initiation and development of dental caries, periodontal diseases, and enhanced development of oral cancers^5,6^. As such, understanding how microbial factors contribute to the establishment and maintenance of oral systems is of great importance, as they can impact the speciation and nature of the oral microbiome. A major constituent of the oral microbiome is the genus *Streptococcus*. Streptococci make up approximately 10-60% of the oral microbiome, depending on the niche examined and sequencing techniques used^3,7,8^. Some of these organisms are keystone colonizers and possess the ability to drastically impact microbial balance^4,9^. Recently, we discovered that *Streptococcus mutans* and *Streptococcus ferus* can alter the growth of other oral streptococcal species via small post-translationally modified peptides called tryglysins^10^. Tryglysins form a new class of post-translationally modified peptides known as “RaS-RiPPs”^11^, with **RaS** and **RiPP** standing for **Ra**dical **S**-adenosylmethionine enzyme and **Ri**bosomally translated and **p**ost-translationally modified **p**eptide, respectively^12^. To date, sixteen different classification groups of streptococcal RaS-RiPP peptides that form unique heterocyclic and macrocyclic structures have been described^11,13^. The tryglysin system in *S. mutans* is induced via quorum sensing and inhibits species such as *S. mitis, S. oralis*, and S*. sanguinis* at concentrations as low as 100 nM^10^. Owing to this potent activity, we wondered what impact these peptides might have on a complex oral system that includes multiple oral species.

Current models of complex oral systems have placed substantial efforts into optimizing culturing conditions. Most frequently used is SHI medium under anaerobic or microaerophilic conditions, replicating growth in a biofilm-like context^14–17^. SHI medium is a nutrient-rich and multi-component medium designed by Edlund et al.^14^, to support the growth of a diverse oral community^14^. Edlund et al. and other investigators have shown that under anaerobic conditions saliva inoculation into SHI medium supports a diverse community^14,18^. These studies have demonstrated that culturing in SHI results in the growth of *Streptococcus*, as well as *Veillonella*, *Gemella*, *Granulicatella*, *Klebsiella*, *Lactobacillus*, and *Fusobacterium*^14^. Anaerobic conditions are primarily used due to the observation that many oral species are strict or obligate anaerobes, and previous studies found that saliva has a low oxygen content^3,4,19^. Biofilms are typically considered to be more relevant to oral systems due to their role in the formation of plaque and establishing oral communities on the dental surface^4,20^. Few investigations have examined the impact of other types of growth conditions, be it the impacts of medium, saliva, oxygen or the type of growth model. In the development of experimental conditions to test tryglysin activity on oral microbes, we found that environmental settings were a critical variable. The impact of culturing conditions on the fungal component of the microbiome is an additional consideration. Fungi are normal constituents of the oral microbiome and have significant effects on oral health^1,21^. However, fungi are less studied in oral systems due to their lower abundance, the difficulty of cultivation, lower sequencing database reliability, and the relative low rate of detection in shotgun metagenomics studies^1,2,21^. Fungal genera growth under anaerobic conditions has typically been observed to be low^16^.

Herein, we describe the first report of tryglysin activity on oral saliva samples. Our initial attempts to observe the impacts of tryglysin on oral microbiota were hampered by barriers associated with culturing conditions. We discovered that culturing in streptococcal chemically defined medium (CDM) was necessary for the activity of the RaS-RiPP tryglysin and that this medium selectively favored the growth of streptococci, even from a rich starting salivary inoculum. We also determined that the type of growth model used and the saliva and oxygen contents had substantial effects on the development of the oral consortia, and that serial culturing led to shifts in the detected species. Finally, we found that 5% CO_2_ allowed for the growth of fungal members of the oral community, making this a culturing method worth considering for future oral studies.

## Results

### Tryglysin A inhibits the growth of saliva-derived oral species in a chemically defined medium that favors *S. salivarius*

Previous studies have demonstrated that tryglysins can inhibit the growth of many streptococcal species^10^. As such, we reasoned that tryglysins should have the capacity to modulate the growth of oral streptococci and potentially the oral microbiome. To examine this aspect further, we first determined if tryglysin A (TryA, Fig. 2D) could inhibit the growth of sensitive streptococci in media typically used for ex-vivo culturing of oral species. One such medium that is frequently used in the oral microbiome field is SHI^14–16,22,23^, and a previous study demonstrated that other synthetic antimicrobial peptides (e.g., C16G2) were effective against streptococci in this medium^24^. To examine activity in SHI, we exposed the TryA-sensitive species *S. mitis*^10^ to an inhibitory level of TryA or a peptide with no known function in quorum sensing or growth (reverse SHP or revSHP) in SHI medium (sequences listed in *Methods*). As SHI medium contains blood and hemin, their inherent turbidity and light-absorbing properties are confounders in optical measurements. As such, viability of microbes was determined by plating for CFU/mL over time (Fig. S1A). To our surprise, SHI medium abrogated any significant inhibition of TryA against *S. mitis* (Fig. S1A). This was not due to a difference in lab-specific strain backgrounds, as retesting with the same strain of *S. mitis* in a separate laboratory setting produced the same results: unobservable activity in SHI medium (Fig. S1B). This was also not due to issues in TryA preparation, as we continually verified that isolated TryA was pure and intact by HPLC and HR-MS/MS (Fig. S2A, B, Table S1). Inactivity in SHI could be due to several reasons: sequestration of TryA, degradation, structural alteration, or potentially excess peptides blocking TryA access to cells. To test if peptide interference was partially responsible for this phenotype, we added the same concentration of peptides as in SHI medium to CDM and observed if TryA was still inhibitory. We found that TryA had reduced activity in this condition, indicating that TryA activity can be blocked by addition of other peptides to CDM (Fig. S2C, D). This implies that at least one reason TryA is inactive in SHI medium is due to increased peptide concentration.

**Fig. 1:**
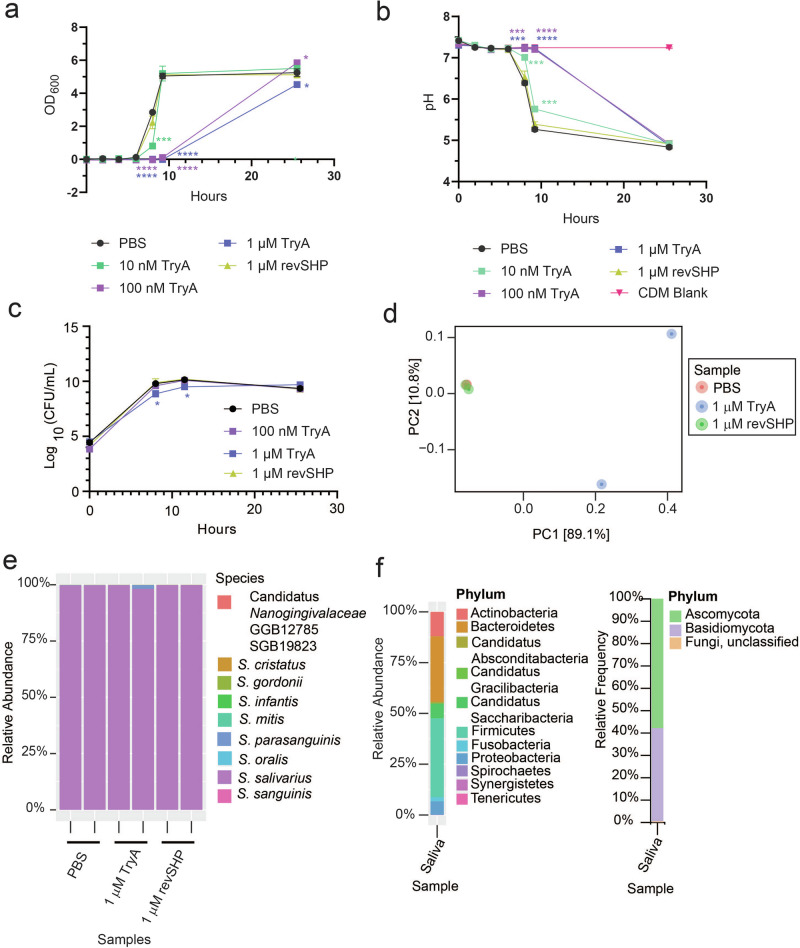
Examination of response of salivary inoculum in chemically defined media (CDM) to the addition of Tryglysin A (TryA), a control peptide that does not affect growth or quorum sensing (reverse SHP/revSHP), or PBS. Concentrations of TryA are indicated in the legend below graphs and correspond to the respective symbol types. Statistical significance compared to PBS control condition as indicated on **a**–**c** was determined using a One-way ANOVA with Tukey’s Multiple Comparisons Post-test; *p < 0.05; ***p < 0.005; ****p < 0.0001. Statistical significance is color-coded to match the corresponding figure legend. **a** Growth curve of salivary inoculum in CDM over time, with statistical significance shown for hours 8 through 24, if values are not indicated for these time points comparisons were non-significant. This experiment was performed three times with similar results. **b** Examination of pH of salivary inoculum in CDM over time, with statistical significance shown for hours 8 through 24, if values are not indicated for these time points comparisons were non-significant. This experiment was performed four times with similar results. **c** Separate experiment examining CFU/mL of salivary inoculum in CDM over time. If statistical values are not indicated, they are non-significant. This experiment was performed twice. **d** Jaccard PCoA plot of metagenomics results from samples presented in **e**. **e** Speciation as determined via shotgun metagenomics for samples grown in the presence of PBS, 1 µM TryA, or 1 µM reverse SHP peptide in CDM for 24 h. Relative abundance is plotted for each sample. Species detected are indicated by the legend beside the graph. **f** Composition of bacterial and fungal phyla detected in human pooled saliva from Norway by shotgun metagenomics and ITS sequencing.

We then proceeded by culturing a previously collected saliva inoculum^16^ in CDM anaerobically^14,15^, since *S. mitis* inhibition was reproducibly observed in this medium. Experimental conditions are outlined in Table 1 and subject demographics for this saliva collection, designated “Norway” are listed in Table 2. We first determined if inhibition was apparent by growing the salivary inoculum in CDM, as well as by measuring pH and optical density over time. The salivary inoculum was placed in CDM and exposed to a range of TryA concentrations, revSHP, or PBS. We observed that TryA delayed growth of the salivary inoculum in a dose dependent manner and this was reflected in the delayed acidification of the medium (Fig. 1A, B), whereas revSHP and PBS treatments grew rapidly (Fig. 1A, B), consistent with previous observations for mono-species cultures of streptococci^10^. CFU/mL was not drastically reduced over time (Fig. 1C, Fig. S1C). We performed 16S rRNA sequencing on these samples after 24 h and primarily observed streptococci (Fig. S1D). To achieve species-level resolution, we carried out shotgun metagenomics and analyzed the data with MetaPhlAn3. From this analysis, we found a high predominance of *Streptococcus salivarius* under all conditions, indicating strong selection for this species after 24 h (Fig. 1E, Supplementary Data 2). Other streptococcal species were observed, but their abundances were low (less than 0.2-2% relative abundance) and varied between the sample conditions (Fig. 1E, Supplementary Data 2). These observed differences were not due to the initial salivary inoculum being skewed in composition, as direct sequencing of saliva showed a diverse composition of phyla and genera of both bacteria and fungi (Fig. 1F). Differential abundance analysis using DESeq2^25^ revealed that TryA treated samples had significantly higher levels of *Candidatus Saccharibacteria* than both PBS and revSHP treated samples (Adjusted *P* < 0.05; Supplementary Data 3). *S. parasanguinis* levels were sensitive to the addition of either TryA or revSHP (Supplementary Data 2-3). No significant differences between samples were observed via alpha diversity metrics such as Chao1 and Shannon (Supplementary Data 4). Principle component analysis (PCoA) analysis of MetaPhlAn3 results showed that TryA treated samples clustered away from other treatment conditions, but these differences were non-significant by PERMANOVA (R^2^ = 0.403, *P* = 0.467) (Fig. 1D). We conclude that CDM leads to strong selection for streptococci under anaerobic conditions, specifically *S. salivarius*. These findings also suggest that TryA may have indirect effects on the presence of oral bacteria such as *Candidatus Saccharibacteria*, organisms that form obligate relationships with other bacteria for their survival^26^. Even though these results were limited due to overgrowth of streptococci in this medium, this demonstrates a slight advantage over single laboratory isolate studies. Typically, single-species models use “domesticated” laboratory strains that have been propagated in culture for years, potentially altering their behavior compared to their in vivo counterparts. Therefore, a model that predominantly consists of streptococci from human saliva could be relevant for understanding species-specific interactions in streptococcal communities, despite its disadvantages in terms of preserving ecological complexity. CDM culturing of saliva represents one such model that could potentially be used. In addition to this, these findings represent the first examination of the potential impact that a streptococcal RaS-RiPP can have on oral systems.

**Table 1:** Experimental parameters used in this study to examine impact of culturing conditions on ex-vivo oral microbiomes

Goal	Number of replicates per experimental condition	Ex-vivo saliva sources	Incubation conditions before sequencing	Community sequencing technique used^a^
Examine impact of tryglysin A on ex-vivo oral microbiomes.	2	Norway	Anaerobic, glucose CDM^b^, 24 h, planktonic^d^	16S sequencing, shotgun metagenomics
Determine impact of pooled human saliva source on ex-vivo speciation.	1 for Norway, 3 for Chicago	Norway, Chicago	No culturing, direct extraction	ITS sequencing, shotgun metagenomics
Examine impact of biofilm vs. planktonic culturing methods.	3	Chicago	5% CO_2_, glucose SHI^c^, 24 h, planktonic or biofilm^d^	16S sequencing, ITS sequencing
Examine impact of CDM vs. SHI medium.	3	Chicago	5% CO_2_, glucose SHI or CDM^c^, 24 h, planktonic^d^	16S sequencing, ITS sequencing

^a^16S sequencing, ITS sequencing, and shotgun metagenomics sequencing were performed as detailed in *Methods*.

^b^CDM stands for streptococcal chemically defined medium.

^c^SHI stands for SHI medium, from Edlund et al.^14^. *Microbiome*.

^d^Planktonic cultures were grown in broth suspension in 15 mL tubes, whereas biofilm cultures were grown as 1 mL cultures in 24 well plates. For further details see *Methods*.

**Table 2:** Subject demographics for saliva collection at University of Illinois - Chicago^a^ and University of Oslo^b^

Demographic	Chicago *N*	Chicago Percent	Norway *N*	Norway Percent
Age group				
18–24	1	12.5%	0	0%^c^
25–34	5	62.5%	0	0%^c^
35–44	1	12.5%	7	87.5%
45–54	1	12.5%	0	0%^c^
>55	0	0%^c^	1	12.5%
Gender				
Male	5	62.5%	2	25%
Female	3	37.5%	6	75%

^a^All data collected at University of Illinois – Chicago for this study was collected at the University of Illinois Chicago CCTS Clinical Research Center, under UIC Research IRB Study ID 2023-0288.

^b^All data collected at University of Oslo for this study was collected at University of Oslo under Norwegian Regional Ethics Committee Study ID REK20152491.

^c^0% indicates no samples were collected from this age group category.

### *S. salivarius* is less sensitive to Tryglysin A treatment and outperforms other oral streptococci in CDM

**Fig. 2:**
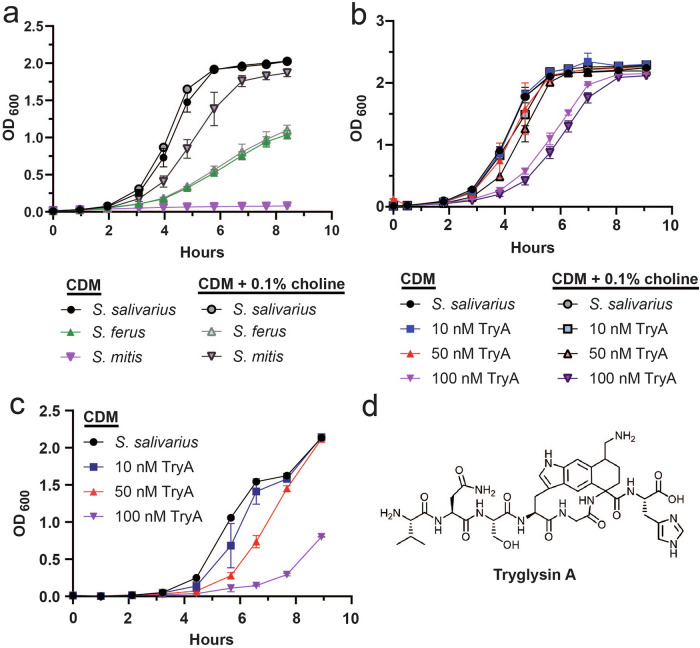
Examination of *S. salivarius* response to Tryglysin A (TryA) in various conditions. Concentrations of TryA are indicated in graph legends and correspond to the respective symbol types. These experiments were performed three times with similar results. **a** Growth curve of wild-type oral streptococci (*S. salivarius, S. mitis*, and *S. ferus*) in CDM or CDM supplemented with 0.1% choline chloride in 5% CO_2_. **b** Growth curve of wild-type *S. salivarius* in 5% CO_2_ exposed to increasing TryA in CDM or CDM supplemented with 0.1% choline chloride. **c** Growth curve of wild-type *S. salivarius* in anaerobic (less than 0.2% O_2_) conditions exposed to increasing TryA in CDM. **d** Structure of TryA.

The observation that *S. salivarius* dominated CDM salivary cultures led us to examine if this species was particularly well-suited for growth in this medium. We examined the growth of several other oral streptococcal species compared to *S. salivarius* in CDM (*S. mitis, S. ferus*). We observed that *S. salivarius* had a faster doubling time (~35 min, Table 3) and reached a higher final OD_600_ in CDM than other tested oral streptococci, at least in conditions such as 5% CO_2_ (Fig. 2a). We also observed that this strain did not require choline for optimal growth, unlike our wild-type *S. mitis* strain (Fig. 2a), and what has been reported for streptococci such as *S. pneumoniae*^27,28^. These data indicate that *S. salivarius* outperforms at least these other streptococcal species in CDM, and that it could outcompete other oral species in co-culture. We did not examine this finding further, but this data indicates that CDM does not meet the metabolic needs of oral streptococci equally. It also corroborates the finding that CDM selects for certain streptococcal species from saliva. This is an important aspect to be aware of using this medium, especially for ex-vivo saliva culturing.

**Table 3:** Doubling times of oral streptococci in CDM or CDM supplemented with 0.1% choline

Species	DT, CDM^a^	DT, CDM + 0.1% choline^a^
*S. salivarius*	35 ± 2	36 ± 0.5
*S. ferus*	93 ± 11	90 ± 7
*S. mitis*	NA^b^	48 ± 3

^a^DT in minutes, plus or minus standard error of the mean.

^b^DT was in excess of 300 minutes, considered as not growing in this condition.

We additionally investigated if *S. salivarius* was inherently more resistant to TryA. Other oral streptococcal species such as *S. mitis* are strongly inhibited for growth at 100 nM TryA^10^. *S. salivarius* was slightly inhibited by TryA at 100 nM in both 5% CO_2_ and anaerobic conditions, but not to the extent observed for other oral streptococcal species (Fig. 2b, c). We noted that TryA was more inhibitory in anaerobic conditions (Fig. 2c). Due to this finding, we examined if the TryA operon was present in sequenced *S. salivarius* deposited to NCBI genome via protein BLAST^29^. We reasoned the presence of the TryA system could provide a potential resistance mechanism. We examined sequenced *S. salivarius* isolates for the presence of the Rgg transcriptional regulator that controls TryA expression and the operon that encodes for its production, called *wgk*. We did not observe the *wgk* operon in the majority of *S. salivarius* genomes on NCBI (accessed June 2025). However, we did identify four sequenced isolates that appeared to possess this system, with high percent identity compared to the *wgk* operon from the producer species *S. ferus* (Table 4). This provides evidence that although few, at least some *S. salivarius* isolates might possess the ability to produce TryA.

**Table 4:** Identification of *S. salivarius* on NCBI genome possessing the *wgk* operon

GCA	Source and assembly level	Source	Location of source	Percent identity vs *wgk* operon from *S. ferus*
GCA_046993525.1	Metagenome, contig^a^	Human infant stool	St. Louis, MO, USA	91.73
GCA_036552945.1	Metagenome, contig	Human stool	Marshfield, WI, USA	91.62
GCA_046885825.1	Metagenome, scaffold	Human infant stool	St. Louis, MO, USA	91.62
GCA_902850305.1	Pure culture, scaffold	Human blood	Besancon, France	91.62

^a^Genome length was reported as too small on NCBI genome.

As a follow-up, we were curious if the *S. salivarius* in our ex-vivo CDM cultures had the *wgk* operon. We performed metagenomic assembly for our samples and annotated the one genome that met minimum metagenomic assembly (MIMAG) standards^30^ for a medium quality draft using BAKTA^31,32^. This was then taxonomically assigned via nucleotide BLAST of the 23S-16S region^29^. Binning statistics, as well as assignment statistics from BAKTA are shown in Supplementary Data 5. This MAG was identified to be *S. salivarius*, and we examined if it had the *wgk* operon using protein BLAST^29^. We observed no significant protein BLAST hits for the Rgg transcriptional regulator or *wgkBC*. We did observe one hit to *wgkD*, but percent identity was low (22.8%) and upon examining its location in the MAG this was not present in a RaS-RiPP operon. From these findings, we believe that *S. salivarius* dominance in CDM and under TryA treatment is likely a combination of the species higher intrinsic resistance to TryA, as well as its ability to grow in CDM.

### Collection of a secondary salivary pool and effects of growth model and oxygen presence

Due to our findings regarding CDM growth with TryA, we decided to investigate how ex-vivo oral culturing conditions could impact these experiments in general. We additionally wished to identify conditions that would allow for more diverse speciation for our assays with CDM or other media, while keeping TryA activity intact. Thus, we collected a secondary saliva pool at the University of Illinois Chicago to determine how parameters such as media composition, presence of CO_2_ and oxygen, initial starting saliva proportions, and growth model affected speciation outcome (Table 1). Subject demographics comparing saliva collections from Chicago and Norway are shown in Table 2. Saliva samples were collected, pooled, and grown under various conditions.

**Fig. 3:**
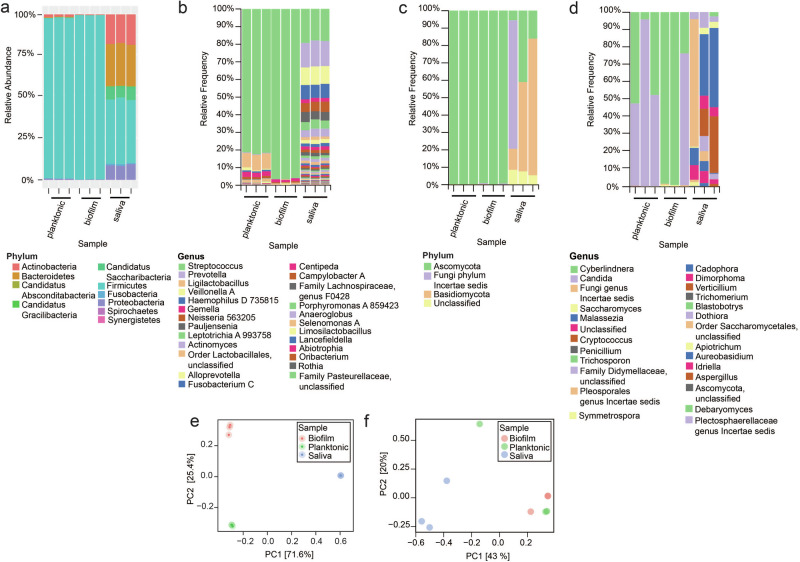
Composition of Chicago saliva collection and examining the use of 5% CO_2_ as a culturing method for ex-vivo oral microbiomes. All samples shown were grown in SHI media for 24 h with 5% CO_2_. **a** Shotgun metagenomics results showing bacterial phyla detected in a biofilm or planktonic model of growth, compared to phyla detected in the original salivary inoculum. **b** 16S sequencing results showing bacterial genera detected in a biofilm or planktonic model of growth, compared to genera detected in the original salivary inoculum. **c** ITS sequencing results showing fungal phyla detected in a biofilm of planktonic model of growth, compared to phyla detected in the original salivary inoculum. **d** ITS sequencing results showing fungal genera detected in a biofilm of planktonic model of growth, compared to genera detected in the original salivary inoculum. **e** Jaccard PCoA of samples in (**a**) from shotgun metagenomics sequencing at the species level. **f** Bray–Curtis PCoA of samples in **c**, **d** from ITS sequencing.

The species composition of the pooled saliva collection from Chicago was compared to the Norway salivary collection. Overall, bacterial phyla and genera detected by shotgun metagenomics were similar between the two collections, aside from the low but measurable presence of an uncharacterized mycoplasma species of *Tenericutes* in the Norway collection not present in the Chicago collection (Figs. 1f, 3a-2b, Supplementary Data 6, 7). Variation between the two collections was present but non-significant, as observed via Jaccard PCoA and PERMANOVA (R^2^ = 0.957, *P* = 0.25) (Fig S3A). We also examined fungal composition by ITS sequencing. This demonstrated that the previous saliva collection was composed primarily of *Ascomycota* and *Basidiomycota*, with the genus *Malassezia* being dominant (Fig. 1f, Fig. 3c, d, Supplementary Data 8). Composition of collections were similar at the phylum level but differences were observed in fungal genera (Fig. 1f, Fig. 3c, d, Fig. S1E). For instance, the Norway collection contained *Penicillium* which was not observed in the Chicago collection, whereas the Chicago collection contained *Cryptococcus* that was unobserved in the Norway collection (Fig. 3d, Fig. S1E, Supplementary Data 8, 9). Further analysis found no significant differences by alpha diversity (Simpson’s Dominance, Shannon Entropy, Chao1 Index and Faith’s) or beta diversity metrics (PERMANOVA: R^2^ = 0.532, *P* = 0.25) (Supplementary Data 10). Bray-Curtis PCoA also reflects these findings (PERMANOVA: R^2^ = 0.446, *P* = 0.5; Fig. S3B). However, this interpretation also relies on the fact that the Norway consortia was only sequenced once due to the limited availability of biomaterial.

We next decided to look at the impact of growth models (biofilm vs planktonic) and oxygen content on bacterial speciation. Culturing techniques used are outlined in Table 1. Chicago salivary samples were grown under 5% CO_2_ and compared to the uncultured, pooled Chicago saliva via shotgun metagenomics, 16S and ITS sequencing, as well as previously published results^14^. We found that the relative frequency of bacterial and fungal phyla decreased in both biofilm and planktonic samples compared to saliva samples (Fig. 3a, c). Both biofilm and planktonic samples were predominated by Firmicutes for bacteria and Ascomycota for fungi, which correlated with *Streptococcus*, *Cyberlindera*, and *Candida* levels respectively (Fig. 3a–d). Bacterial and fungal genera were detected and a summary of the ASV counts from QIIME2 analysis and relative abundance of shotgun metagenomics analysis are listed in Supplementary Data 7, 9, 11. Alpha diversity metrics for biofilm and planktonic samples were significantly different for 16S sequencing results (Fig. S3C), but not ITS sequencing (Fig. S3D). Beta diversity demonstrated that for both 16S and ITS sequencing, compositions of samples were distinct (Fig. S4A-S4B). PCoA and PERMANOVA indicated that samples grown in a biofilm versus planktonic model were significantly different from each other, and distinct from the salivary inoculum (R^2^ = 0.993, *P* = 0.002 for shotgun metagenomics; R^2^ = 0.449, *P* = 0.018 for ITS) (Fig. 3e, f). We note that these biofilm samples were not grown in plates with a pre-treated saliva coating, which marks one additional change besides the culturing in 5% CO_2_ compared to previous culturing studies^14–16^. However, we find that like the previous Edlund et al.^14^ study using the pre-coated saliva method, streptococci are predominant, although the relative percentage of streptococci increases under 5% CO_2_ without a saliva coat (Fig. 3b, Supplementary Data 12). In planktonic samples, streptococci make up 81.8% of genera detected (Fig. 3b, Supplementary Data 12). *Veillonella, Gemella, Granulicatella, Lactobacillus, Prevotella, Porphyromonas, Neisseria* were also detected in our study, though at lower proportions than reported in Edlund et al. More bacterial genera were detected in the planktonic samples in our study than in either biofilm culturing methods (Supplementary Data 12). Additionally, we observed that samples cultured at 5% CO_2_ had observable levels of fungi. Previous studies have observed fungal levels using a pre-coated saliva biofilm model with SHI medium to be low during anaerobic culturing^16^. In contrast, our samples were dominated by detectable *Cyberlindnera* during biofilm culturing and *Cyberlindnera* and *Candida* during planktonic culturing (Fig. 3d, Supplementary Data 9).

Overall, these data indicate that although not perfect, culturing saliva consortia with 5% CO_2_ encourages the growth of oral streptococci alongside other major oral genera and allows the propagation of certain fungi. This represents a much more accessible method of culturing oral microbiota than prior methods, as it does not necessitate an anaerobic chamber. It also indicates that this method could provide a means to assess oral fungal-bacterial interactions.

### Impact of media conditions and re-culturing on consortia results

**Fig. 4:**
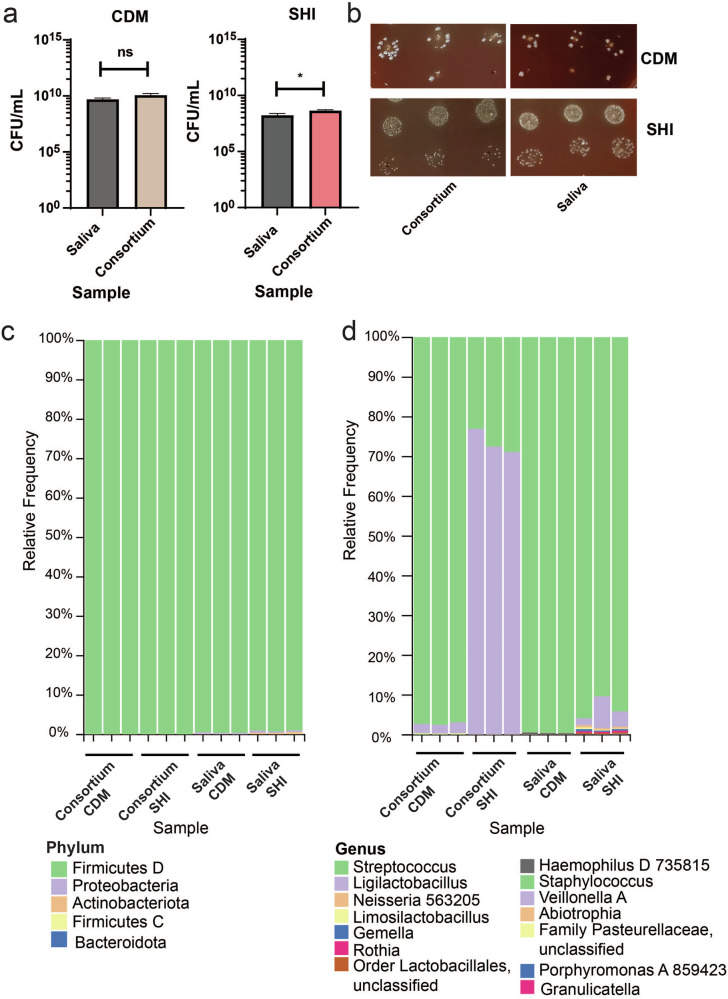
Examining the impact of CDM vs. SHI medium on culturing ex-vivo oral microbiomes. Samples were grown in CDM or SHI medium for 24 h with 5% CO_2_, in biological triplicate. The term “consortia” indicates saliva samples that were previously grown in SHI medium and stored for further propagation. **a** CFU/mL of salivary inoculum or previously stored consortia after growth in CDM or SHI medium. Statistical significance was determined using an unpaired two-tailed *T*-test; *p < 0.05; ns, nonsignificant. These experiments were performed twice with similar results. **b** Colony morphologies observed for saliva or consortium propagation in CDM or SHI medium after 24 h of growth. This experiment was performed twice with similar results. **c** 16S sequencing results showing bacterial phyla detected after growth of saliva inoculum or previously stored consortia in CDM or SHI medium. **d** 16S sequencing results showing bacterial genera detected after growth of saliva inoculum of previously stored consortia in CDM or SHI medium.

We determined that culturing the Norway salivary sample in CDM led to consortia collapse to streptococci (Fig. 1e), To further examine this finding, we tested whether selection would be similar with the Chicago consortia using the 5% CO_2_ planktonic model with CDM or SHI medium, as well as to examine the impact of serial culturing on species diversity. SHI medium cultures grown for 24 h were frozen at a minimum of −70 °C as glycerol stocks and then thawed, split, and used to inoculate fresh SHI or CDM media as triplicate samples for 24 h and then examined for their bacterial and fungal composition. Total CFU/mL displayed no differences between serially cultured samples or cultured saliva in CDM, but CFU/mL was higher in serially cultured samples in SHI medium (Fig. 4a). No major differences in colony morphologies were observed between conditions (Fig. 4b).

**Fig. 5:**
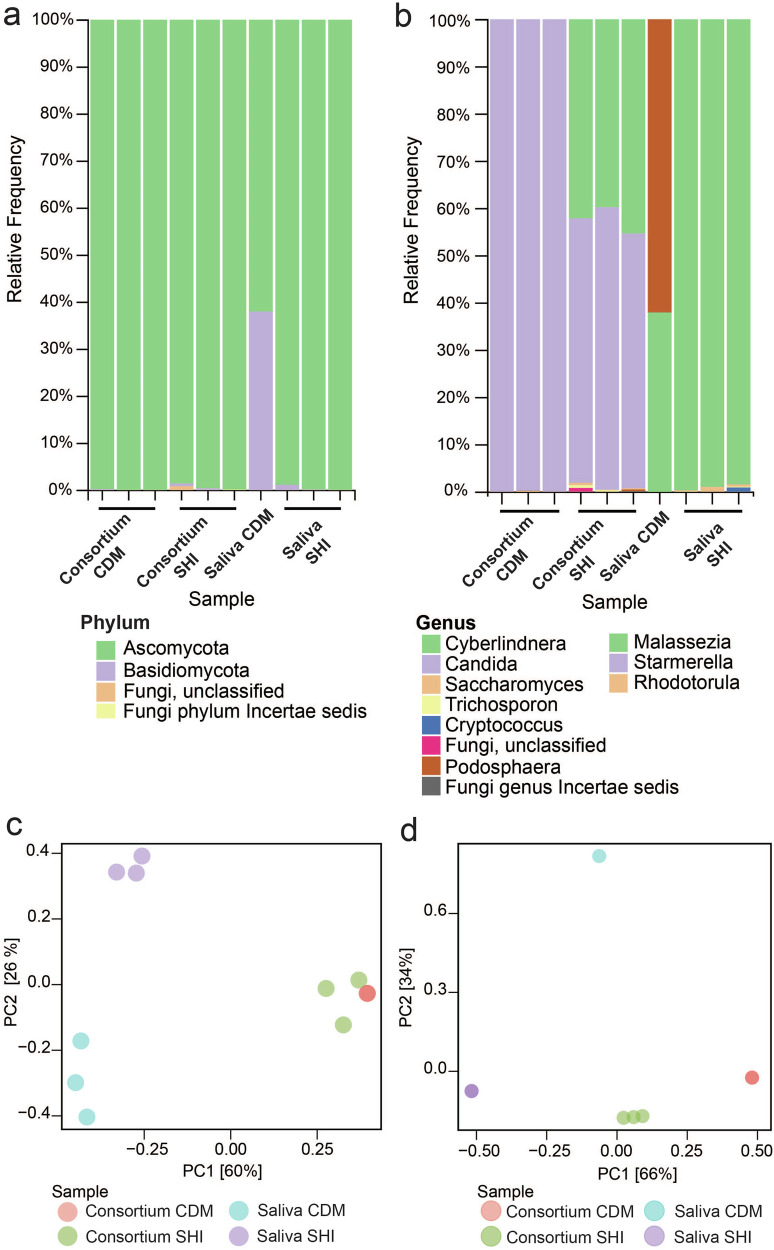
Additional data examining the impact of CDM vs. SHI medium on culturing ex-vivo oral microbiomes. Samples were grown as outlined in Fig. 4. The term “consortia” indicates saliva samples that were previously grown in SHI medium and stored for further propagation. **a** ITS sequencing results showing fungal phyla detected after growth of saliva inoculum or previously stored consortia in CDM or SHI medium. **b** ITS sequencing results showing fungal genera detected after growth of saliva inoculum or previously stored consortia in CDM or SHI medium. **c** Jaccard PcoA of samples in Fig. 4c, d from 16S sequencing. **d** Bray–Curtis PcoA of samples in panels A-B from ITS sequencing.

We then examined bacterial composition by sequencing and made several important findings. We observed that the use of serially cultured samples selects for specific bacterial genera in both CDM and SHI medium: *Streptococcus* and *Ligilactobacillus* (Supplementary Data 13, Fig. 4c, d). The relative amounts of these change depending on the medium condition, with SHI selecting for more *Ligilactobacillus* and CDM selecting for more *Streptococcus* (Fig. 4d; Supplementary Data 13). In contrast, direct culturing of saliva in SHI medium leads to a higher number of bacterial genera detected than in CDM (total of 13 vs. 8). We also found that culturing saliva directly in CDM led to the growth of primarily streptococci (Fig. 4d; Supplementary Data 13), similar to the results from our initial investigations (Fig. 1e). These differences are reflected in the alpha and beta diversity analysis of these results (PERMANOVA: R^2^ = 0.876, *P* = 0.001) (Fig. S5-S6). For alpha diversity, Simpson’s Dominance and Shannon Entropy were statistically significant between CDM and SHI medium, with SHI medium having higher relative values for these metrics (Fig. S6A) indicating that species richness, relative abundance, and relative evenness are higher for samples cultured in SHI medium. In contrast, Faith’s Phylogenetic Diversity and Chao1 Index were only significant when comparing the use of inoculum source (Fig. S6A). This indicates that genera richness decreases when using re-cultured consortium as a source. Finally, beta diversity examined using Jaccard distance, and was significantly different when comparing inoculum sources only, indicating that this condition is key for determining the number of unique species (PERMANOVA: R^2^ = 0.572, *P* = 0.003) (Fig. 5c, S5B, S6B). This heavily suggests that continually re-cultured samples in SHI medium should be used with caution for oral microbiome studies, as a clear skew in genera representation results.

We then compared fungal composition from the same samples. Similar to previous results for planktonic culturing in SHI medium, *Ascomycota* was the dominant phylum (Figs. 3c, Fig. 5a, Supplemental Data 14). Continued culturing in either medium led to predominance of *Candida* (Fig. 5b, Supplemental Data 14), indicating that re-culturing leads to a selection for specific fungal genera. No pairwise differences in alpha or beta diversity via Bray-Curtis were observed between samples (Fig. S7). Overall microbiome composition was significantly different between groups (PERMANOVA, R^2^ = 0.997, *P* = 0.001) (Fig. 5d). Significant differences were observed in alpha and beta diversity when examining medium conditions alone (PERMANOVA, R^2^ = 0.543, *P* = 0.005) (Fig. S8A-S8B). Inoculum source was also significant when compared alone (PERMANOVA, R^2^ = 0.449 *P* = 0.015) (Fig. S8B). This indicates that the differences in re-culturing and media composition of fungi are significant for fungal species diversity. However, this analysis could also be affected by the lower number of fungal reads obtained for this experiment and should be interpreted with some caution.

## Discussion

Our initial investigations started by examining the impact of tryglysin A on the growth of an ex-vivo oral system. Initially, we found that TryA was ineffective in SHI medium (Fig. S1A), prompting us to switch to culturing in CDM, a peptide free medium known to support TryA activity^10^ (Fig. 1). This phenomenon, where peptides show activity in minimal but not rich media, has been documented in certain cases^33–35^. Small peptides have been demonstrated in other systems to gain access to cells via transporters^36,37^, and we believe that TryA may exert its effects via a similar mechanism. This lack of activity in SHI could be multifactorial: binding of TryA by SHI components, degradation, or decreased ability of TryA to access cells. Our experiment examining TryA activity in CDM with SHI peptides provides evidence that this inhibition is at least partially due to competition with other peptides for cellular access (Fig. S2). Future experiments will examine other lines of reasoning as to why TryA is ineffective in SHI, as well as why increased peptide levels partially block TryA activity.

We observed that in CDM TryA significantly affected the presence of *S. parasanguinis* and a member of *Candidatus Saccharibacteria*, suggesting that TryA might be capable of modulating certain members of the oral microbiome (Fig. 1, Supplementary Data 3). However, these conclusions are limited by the fact that we cannot conclude if the effect of TryA is direct or indirect from this data, and that CDM led to a collapse of the consortia to primarily streptococci. These findings will be explored in future work examining TryA effects on *S. parasanguinis* and if observed effects on *Candidatus Saccharibacteria* are due to disruption of their associated helper species or direct inhibition. Additionally, in CDM culturing experiments, we observed that *S. salivarius* emerges as the primary species (Fig. 1). While this limited the findings we could conclude from treatment with TryA, it raised several important questions surrounding *S. salivarius* growth in CDM and TryA resistance. We found that this species is less sensitive to TryA treatment and grows better in CDM compared to other tested oral streptococci (Fig. 2). We did not identify why CDM supported *S. salivarius* growth better than other species, but we hypothesize that this medium uniquely supports this species’ metabolic needs or simply lacks components necessary for the optimal growth of other streptococcal species. We also identified that although type strains of *S. salivarius* do not have the TryA production system, clinical samples do (Table 4). We find that *S. salivarius* in our ex-vivo studies likely did not possess this system, indicating that its dominance in CDM was likely due to intrinsic resistance or CDM’s ability to support its growth. These findings represent the first observation of the potential for streptococcal RaS-RiPPs to influence their residing oral systems, while examining why *S. salivarius* emerged as a primary species in CDM salivary cultures.

In the second part of our study, we aimed to further explore conditions that could influence outcomes in ex-vivo models of the oral microbiota. Previous studies of oral consortia have primarily been performed in anaerobic chambers with atmospheres of varying mixtures of carbon dioxide, nitrogen, and hydrogen^14–16,22^. These conditions allow for the excellent growth of strict anaerobes found in the oral microbiome. However, this does not mean that other culturing methods should not be explored for their efficacy, as other studies have supported the idea that an oxygen gradient occurs in the oral microbiome^3,38^. Saliva has been traditionally considered a low oxygen environment due prior reports of salivary oxygen levels around 0.08 ppm and rapid turnover of oxygen by oral organisms^19,39^. However, a new study by Podunavac et al., found drastically varying levels of oxygen and carbon dioxide between conditions and human volunteers^40^, although this could be partially attributed to sampling methods. Additionally, data from Sikdar et al. ^41^ supports the idea that 5% CO_2_ is a viable culturing method for ex-vivo oral systems^41^. In their study, they found that a supragingival plaque system only produced n-acyl homoserine lactone signaling molecules under the presence of 5% CO_2_, and that these significantly impacted the oral system^41^. Also similar to our findings, they found that under 5% CO_2_ in both a planktonic and biofilm type model *Streptococcus* predominated^41^. This study together with our data indicates that 5% CO_2_ should be considered as a secondary culturing method for oral samples. This additionally makes culturing oral microbiomes more accessible to other laboratories, as anaerobic culturing requires an expensive investment in equipment that limits oral microbiome experiments to those who possess these set-ups.

Culturing in 5% CO_2_ had a clear impact on bacterial phyla and speciation, strongly selecting for Firmicutes (Fig. 3a), but also allowing the growth of fungal species (Fig. 3c). Anaerobic culturing of ex-vivo systems have been reported to have low fungal presence^16^ but we observe that during 5% CO_2_ culturing in SHI medium fungi are present (Fig. 3c, d). This is not dependent on the growth model used, as we observed fungi were present in both a biofilm and planktonic model (Fig. 3c, d). This presence of fungi was dependent on the use of SHI medium, as the use of CDM resulted in fewer fungal ASVs by ITS, and some samples generated few reads (Fig. 5b, Supplementary Data 14). Re-culturing of saliva appeared to select for the presence of specific genera of fungi such as *Cyberlindnera* and *Candida* (Fig. 5b). The underlying reason for this growth of fungi could be due to several reasons. SHI medium under 5% CO_2_ atmosphere could be uniquely suited to the growth of fungi from saliva, or alternatively, the reduction of bacterial diversity in this condition could allow fungi to establish a foothold in this culturing condition. These are lines of investigation worthy of further study. We assert that due to these data and findings concerning other important factors such as AHL production^41^, this culturing method should be used to examine additional aspects of oral microbiome interactions.

In our culturing experiments, we observed differences between growth models at the bacterial and fungal genera level (biofilm vs. planktonic) (Fig. 3). In our initial experiments, a larger salivary inoculum was used for planktonic samples (Fig. 3). As such, the difference between higher saliva concentration and mode of growth cannot be separated. One thing it does emphasize is the fact that both saliva concentration and mode of growth contribute to ex-vivo speciation outcome. This is not surprising, as other studies have found human saliva can greatly alter growth and behaviors of oral species^42,43^. We also observed clear impacts of re-culturing samples from SHI medium. Originally, we hypothesized that SHI could lead to stable and re-culturable consortia for use in the laboratory. However, we found that there were clear impacts at the genus level for both bacteria and fungi for re-culturing (Figs. 4–5). Re-culturing might be a viable method for selecting for simpler fungal and bacterial consortia, but it clearly results in certain biases. Further study is necessary to determine if these conditions could establish stable, simplified microbial models for experimental use.

From this study, we conclude that multiple factors must be carefully considered in ex-vivo oral culturing studies, including the role of atmosphere, culturing method, initial inoculum, and appropriate medium conditions. Optimizing these aspects is essential for developing robust ex-vivo oral models to better understand physiological aspects of the oral microbiome. Notably, while CDM supported fewer species compared to the rich SHI medium traditionally used, it provided insights into TryA activity and how this medium supports streptococcal growth. These findings also suggest that future studies should incorporate examining TryA in a medium condition which provides a more biologically relevant context—such as sterile-filtered saliva. This was a limitation of the current study but could easily be incorporated into future investigations. Sterile-filtered saliva would provide a setting for testing TryA activity while better preserving species diversity. These findings and considerations highlight the importance of selecting the appropriate medium to achieve specific peptide activity or antimicrobial agents in general. The phenomenon wherein certain peptides are functional in a defined medium but not in a rich medium remains an intriguing question. In the meantime, it is important to explore diverse conditions for studying various compounds that may exhibit activity under different circumstances, thereby expanding our understanding and potentially uncovering new therapeutic applications.

## Methods

### Bacterial strains and growth conditions for strains and ex-vivo oral samples

Bacterial strains used in this study are listed in Supplementary Data 1: Table S2. Strains and ex-vivo oral microbiota samples from saliva were grown on Todd-Hewitt plates (TH, BD Biosciences) with 1.4% Bacto-Agar (BD Biosciences) and 0.2% Yeast Extract (VWR, RPI International), Tryptic Soy plates (TS, BD Biosciences) with 1.4% Bacto-Agar (BD Biosciences), Tryptic Soy plates with 1.4% Bacto-Agar (BD Biosciences) and 5% defibrinated sheep blood (QuadFive or Fisher Scientific), in Tryptic Soy Broth (TSB, BD Biosciences), in Todd-Hewitt (TH, BD Biosciences) with 0.2% Yeast Extract (VWR, RPI International), in SHI medium, or in streptococcal chemically-defined medium (CDM) plus 1% glucose at 37 °C with 5% CO_2_. The components and recipe for streptococcal CDM used were as described previously^37^. The components and recipe for SHI medium used were as described previously^14,16^.

### Purification of tryglysin A peptides and synthesis of *S. mutans* reverse SHP peptides

*S. mutans* reverse SHP peptide (GGGIIIITE) was purchased from ABClonal (Woburn, MA. Purities of peptide preparations used in assays were greater than 70%. *S. mutans* reverse SHP peptide was reconstituted as 10 mM stocks in DMSO and was stored in aliquots at −20 °C. For experiments with *S. mutans* reverse SHP peptide subsequent dilutions for working stocks (1 mM, 100 µM, 10 µM) were made in DMSO or PBS and stored at −20 °C.

For purification of tryglysin (VNSWGKH, modification at WGK), *S. ferus* DSM 20646 was grown overnight in 10 mL of Todd Hewitt broth +0.2% yeast extract (5% CO_2_, 37 °C) for 12–15 h. The overnight culture was then centrifuged for 10 min at 4000 × *g*, and the cell pellet was resuspended in 10 mL of CDM. The resuspended bacterial pellet was used to inoculate one liter of CDM in a glass bottle. This culture was then incubated for 20–24 h at 37 °C with 5% CO_2_. The resulting culture was centrifuged for 10 min at 4000 × *g*, and cell-free supernatant filtered through 0.2 µm pore filters (VWR). 500 mL of the resulting filtered supernatant was loaded onto a 1 g HyperSep Hypercarb SPE cartridge (Thermo Scientific, 60106-404), which had been preconditioned with 5–10 column volumes of 100% acetonitrile (ACN) with 0.1% formic acid (FA) followed by 5–10 column volumes of 100% H_2_O + 0.1% FA. The column was then washed with five column volumes of 100% H_2_O + 0.1% FA. Tryglysin A was eluted with 10–20 column volumes of 50% ACN + 0.1% FA. The eluted material was then evaporated to dryness and resuspended in 1 mL of H_2_O + 0.1% FA. Tryglysin A was purified further through two rounds of HPLC using an Agilent 1260 infinity system. In the first round, the resuspended samples were run on a semi-preparative scale with 100 μL injection volumes onto a Phenomenex Luna Omega Polar C18 100 Å column (5 μm, 250 × 10 mm). Compounds were eluted using an initial 3.5-min isocratic step running 100% H_2_O + 0.1% FA followed by a ramp to 10% ACN + 0.1% FA for 5 min, and a final ramp to 100% ACN + 0.1% FA over 2 min. During this time, 0.5 min fractions were collected. Fractions containing tryglysin A were identified through high-resolution QTOF-MS analysis using an Agilent 6540 UHD mass spectrometer. Fractions containing tryglysin were further purified through analytical HPLC with a Phenomenex Luna Omega Polar C18 100 Å (1.6 μm, 150 × 2.1 mm) column using the same method. Several liters of culture were worked up in this manner to obtain sufficient tryglysin yields for experiments. Example HPLC trace data from purified tryglysin and confirmatory MS/MS are shown in Fig. S2 and Table S1, respectively.

### Collection of salivary inoculums

For saliva samples used at the University of Oslo, samples (also designated ‘Norway’ collection) were collected as outlined in Dornelas-Figueria et al. ^16^. These samples were collected in accordance with the Declaration of Helsinki and approved by the Norwegian Regional Ethics Committee (REK20152491) for studies involving human samples. For saliva samples used at the University of Illinois Chicago, samples (also designated ‘Chicago’ collection) were collected in conjunction with the CCTS Clinical Resources Center at the University of Illinois Chicago, in accordance with the Belmont Report and DHHS regulations 45 CFR Part 46 and approved by the University of Illinois Chicago Institutional Review Board (STUDY2023-0288). Sample collection, consent, demographic data collection, and de-identification prior to use by researchers for this study was conducted by CCTS Clinical Resources Center. Subjects were those over the age of 18, not currently using tobacco products, not under treatment with antibiotics or systemic disease, not recently diagnosed or having active COVID-19 infection, considered in good general health, and having good oral health (i.e., not currently having gum disease, excessive cavities, and possessing at least 20 teeth, in other words, not having undergone multiple extractions of teeth). Conglomerate demographics for subjects are listed in Table 2. Subjects were asked to expectorate 5 mL of saliva directly into a collection tube, after which samples were stored at 4 °C. The same day of collection, samples collected and centrifuged at 2600 × *g* for 10 min to spin down large debris and eukaryotic cells, and resulting saliva supernatants were pooled together. Pooled saliva was stored with 20% glycerol at a minimum of −70 °C, until all samples had been collected, at which time they were pooled again and then aliquoted into 1 mL volumes, which was referred to as pooled saliva. Pooled saliva was stored at a minimum of −70 °C for use in experiments.

### Assays examining exposure of *S. mitis* and *S. salivarius* to tryglysin A, and growth curves of oral streptococci in CDM

For examining *S. mitis* or *S. salivarius* response to tryglysin A, pre-stored glycerol stocks or overnight cultures grown in THY were centrifuged and resuspended in 1 mL of CDM or SHI medium. Cultures were centrifuged again at 10,000 × *g* for 5 min, and then washed with 1 mL of CDM, CDM + 0.1% choline (choline chloride), or SHI medium. Cultures were then resuspended in 1 mL of either CDM, CDM + 0.1% choline, or SHI medium and diluted with according medium to OD_600_ ~ 0.03 for *S. mitis* experiments and ~0.01 for *S. salivarius* experiments. For growth curves in CDM or CDM + 0.1% choline, 10 nM, 50 nM, or 100 nM TryA were added to cultures. Samples were grown at 37 °C with 5% CO_2_ or anaerobically (less than 0.2% O_2_), with OD_600_ observed every 45 min to 1 h with a spectrophotometer. Resulting data from growth curves was plotted with Graph Pad Prism 10.5.0 (GraphPad Software, LLC). For CFU/mL assays in SHI or CDM medium, cells were prepared as outlined above and inoculated into 96 or 24 well plates. CDM medium was prepared as previously detailed or with the addition of peptides equivalent to that present in SHI. For each condition in triplicate, no peptide, 1 µM or 10 µM tryglysin A or revSHP (*S. mutans* reverse SHP peptide) were added to wells. Cultures were then incubated at 37 °C with 5% CO_2_. Starting at time 0 (inoculation time after peptide addition), a portion of each sample was transferred to a separate 96 well plate, diluted for CFU/mL in TSB or sterile PBS and plated on either TSA or TSA plus 5% defibrinated sheep blood in technical triplicate for CFU/mL determination. Plates were then incubated at 37 °C with 5% CO_2_ for 1–2 days and colonies enumerated. Resulting CFU/mL for each condition was plotted using Graph Pad Prism 10.5.0, and examined for statistical significance between conditions at each time point via a One-way ANOVA with Tukey’s Multiple Comparisons Post-test. For examining the growth of oral streptococcal strains, strains listed in Table S2 were grown as overnight cultures and strains were grown as previously detailed in CDM or CDM + 0.1% choline, and growth curves plotted and analyzed in Graph Pad Prism 10.5.0 as detailed above.

### Ex-vivo saliva experiments

For growth curves, pH, and CFU/mL assays of salivary inoculum with tryglysin A: Norway collection saliva stocks at a minimum of −70 °C were thawed and inoculated into anaerobically incubated CDM at a concentration of 2 µL/mL in sterile 15 mL tubes. For each condition in duplicate, 10 nM, 100 nM, 1 µM tryglysin A or *S. mutans* reverse SHP peptide (revSHP) or equivalent volume of PBS was added to cultures. For growth curves and pH measurements, cultures were grown in an anaerobic chamber at 37 °C for 24 h. Samples were taken at 0, 2, 4, 6, 8, 10 and 24 h, and measured for growth via observation of OD_600_ and pH. Resulting pHs and growth curve data were plotted using Graph Pad Prism 10.5.0. pH measurements were examined for statistical significance between conditions at each time point via a One-way ANOVA with Tukey’s Multiple Comparisons Post-test. For CFU/mL assays, samples were grown in the same manner with the exception that samples were taken at 0, 8, 12, and 24 h. Samples diluted in sterile PBS at each time point and plated on either TSA or TSA plus 5% defibrinated sheep blood in technical triplicate. Plates were then incubated at 37 °C in the anaerobic chamber 24 to 48 h and colonies enumerated. Resulting CFU/mL values were plotted using Graph Pad Prism 10.5.0, and examined for statistical significance between conditions at each time point via a One-way ANOVA with Tukey’s Multiple Comparisons Post-test. For sample harvest for sequencing, 1 mL of duplicate samples exposed to 1 µM tryglysin A, 1 µM revSHP, or equivalent volume of PBS were harvested at 24 h post growth and stored with 20% glycerol at −20 °C. For direct sequencing of saliva, (i.e., initial inoculum sample), 1 mL of saliva was centrifuged at 14,000 × *g* for 5 min. Supernatant was removed and remaining pellet was processed using the Masterpure Gram Positive DNA Purification Kit (Biosearch Technologies, MGP04100) according to manufacturer’s instructions. Samples and DNA (from bacteria and fungi from initial saliva) were then shipped on dry ice to the University of Illinois Chicago, under University of Illinois Chicago Institutional Review Board approval STUDY2023-0671, not human research determination; and CDC PHS Permit Number 20230524-2079 A. Samples were thawed and extracted according to the manufacturer’s protocols using a Masterpure Gram Positive DNA Purification Kit (Biosearch Technologies, MGP04100). Sample purity was measured using a nanodrop (Thermo Fisher Nanodrop 2000 Spectrophotometer), samples acceptable for sequencing were those with an A28/A260 of 1.8-2.0 and an A230/A260 of approximately 2.0. A minimum of 200 ng of total sample with a minimum concentration of 10 ng/µL were then submitted to the RUSH Genomics and Microbiome Core Facility (RUSH University) for shotgun metagenomics, ITS amplicon sequencing (ITS1F to ITS2R, fungal ITS1 region), and 16S sequencing (515 F and 806 R, bacterial 16S rRNA region). Sample data was processed as outlined in *Shotgun metagenomics sequencing and data processing* and *ITS and 16S amplicon sequencing and data processing*.

For growth and harvest of salivary samples in planktonic or biofilm models with 5% CO_2_ for sequencing and CFU/mL analysis: Saliva samples from Chicago collection stored at a minimum of −70 °C were thawed and inoculated into SHI medium in triplicate in the following manner. For planktonic samples, 2 mL of salivary inoculum was added to 3 mL of SHI medium in 15 mL conical tubes. For biofilm samples, a 1:100 dilution of saliva was added to SHI medium and aliquoted into 1 mL volumes into a 24 well plate. Samples were then grown at 37 °C with 5% CO_2_ for 24 h. At 0 h (inoculation time) and 24 h, a portion of each sample in triplicate was transferred to a separate 96 well plate, diluted for CFU/mL in TSB or sterile PBS, and plated on TSA plus 5% defibrinated sheep blood in technical triplicate for CFU/mL. Plates were then incubated at 37 °C with 5% CO_2_ for 1–2 days, colonies enumerated, and plates imaged. Resulting CFU/mL for each condition was plotted using Graph Pad Prism 10.5.0, and examined for statistical significance between conditions at each time point via a One-way ANOVA with Tukey’s Multiple Comparisons Post-test. At 24 h in the experiment, 1 mL each of cultures were also stored at −20 °C for downstream DNA extraction. Additionally, 1 mL of each culture in duplicate at 24 h were stored with 20% glycerol at a minimum of −70 °C as “cultured consortia” for use in downstream experiments. Therefore, cultured consortia represented salivary inoculum that was previously cultured in SHI medium and stored for further propagation. For DNA extraction, a 3 mL initial saliva sample was prepared and sequenced as well as 1 mL each of experimental samples at 24 h, in the same manner as outlined in previous experiments. Sample data was processed as previously mentioned.

For growth and harvest of salivary samples or re-cultured consortium in CDM or SHI media with 5% CO_2_ for sequencing and CFU/mL analysis: Saliva samples from Chicago collection or “cultured consortia” (saliva inoculum previously cultured in SHI medium) stored at a minimum of −70 °C were thawed and inoculated into SHI medium or CDM in the following manner. Each condition was grown in triplicate. Samples were diluted 1:100 into 6 mL of SHI or CDM, and then incubated for 24 h at 37 °C with 5% CO_2_. At 0 h and 24 h, initial inoculum or cultures were harvested, plated for CFU/mL, and results analyzed in Graph Pad Prism as outlined previously, with the exception an unpaired two-tailed *T-*test was used to determine statistical significance between CFU/mL levels of cultured saliva and consortium. At 24 h, 1 mL each of cultures were also stored at −20 °C for downstream DNA extraction, and samples processed and underwent ITS and 16S sequencing as previously mentioned.

### Shotgun metagenomics sequencing and data processing

Shotgun metagenomic sequencing libraries were prepared with Illumina DNA Prep kit (Illumina, 20060059) then sequenced on NovaSeq X with 2 × 150 bp amplification. Resulting sequencing data for shotgun metagenomics was then submitted to the RUSH Bioinformatics Core (RUSH University). Raw data was checked using FastQC (https://www.bioinformatics.babraham.ac.uk/projects/fastqc/), followed by quality filtering and trimming using the algorithm bbduk (http://jgi.doe.gov/data-and-tools/bb-tools/). Short read taxonomic annotation was performed using the software package MetaPhlAn3 (v4.0.1)^44^ and functional gene annotation with HUMAnN3 (v3.5)^44^ mapping to the UniRef90 catalog (UniRef release 2019_01). UniRef90 relative abundance tables were regrouped into higher-level organizations, including: MetaCyc pathways, KEGG orthology, and UniProt gene families for downstream analysis. Resulting data, such as MetaPhlAn3 taxonomy assignments and BIOM files were imported into R Studio ver. 2024.09.0 + 375 (Posit Software, PBC) and data was processed using the R packages dplyr and tidyr^45,46^, to generate a stacked bar plot for relative abundances using the ggplot2 package^47^. PCoA plots were generated from MetaPhlAn taxonomic abundance data imported into R Studio and processed using the R packages metaphlanToPhyloseq, phlyoseq, and dplyr^46,48,49^. Ordination plots for PcoA were generated from taxonomic abundance data using the ordinate function in the phyloseq package with the PCoA method and Jaccard distance settings. PCoAs plots were plotted using ggplot2 and phyloseq packages in R Studio, with the imported MetaPhlAn3 taxonomic abundance data and ordination plots generated by the phyloseq package. Total read counts obtained from bbduk quality filtering and trimming were then used to estimate the relative count values based on MetaPhlAn3 taxonomic abundance data. These relative count values were used to perform statistical analysis of MetaPhlAn3 taxonomic abundance using DESeq2^25^. For analysis of shotgun metagenomics alpha diversity metrics, the relative count values based on MetaPhlAn3 taxonomic abundance data were analyzed with the estimate_richness function in the phyloseq package in R Studio. Resulting values from alpha diversity metrics were plotted using Graph Pad Prism 10.5.0, and examined for statistical significance between conditions using a via a Kruskal–Wallis pairwise test with a Benjamini & Hochberg correction^50^. Statistical significance of beta diversity metrics via PERMANOVA (adonis function)^51^ was determined using the vegan R package and pairwiseAdonis function^52^. We did note that for shotgun metagenomics samples grown in SHI medium, a large number of reads from *Ovis aries* were observed in these samples, which were filtered out during the shotgun metagenomics bioinformatics analysis. This correlates with the use of sheep blood during SHI medium culturing.

Before deposition of metagenomics sequence results to NCBI SRA, sequences were screened and filtered for potential human host sequences using Bowtie2^53^ with the human genome GRCh37 (NCBI RefSeq accession: GCF_000001405.13). Additional human read scrubbing was also performed by the Sequence Read Archive. All raw sequence reads for experiments are deposited to the NCBI SRA under BioProject PRJNA1189147.

### Identification of the tryglysin operon *wgk* in *S. salivarius* and examination of *S. salivarius* from shotgun metagenomics experiments

To determine if *S. salivarius* possessed the biosynthetic operon for tryglysin (*wgkABCD*)^10^, the previously published protein sequences for WgkB and the regulatory Rgg PdrA were input into nucleotide and protein NCBI BLAST, respectively^29^. Default parameters were used for the search, with the parameter for Organism set to *Streptococcus salivarius* (taxid: 1304). This identified proteins of high percent identity, to which the matching nucleotide sequence and surrounding genes were compared to the full *wgk* operon. To confirm high similarity, nucleotide region corresponding to the *wgk* operon from *S. ferus* was compared to each identified putative operon from *S. salivarius* using Clustal Omega^54^.

To examine if the *S. salivarius* from the CDM salivary experiments with TryA possessed the operon: shotgun metagenomics data generated as described previously were quality filtered and trimmed for Nextera adaptor sequences using Trimmomatic (v. 0.39)^55^, filtered for potential human host sequences using Bowtie2^53^ with the human genome GRCh37 (NCBI RefSeq accession: GCF_000001405.13), assembled using metaSPAdes (v. 3.15.5)^31^ with default parameters. Resulting assembly contigs were indexed, mapped, and read depth files were generated using Bowtie2 and MetaBAT2^56^. These data were binned using MaxBin 2.7.7^57^ and resulting MAGs were examined for quality using CheckM^58^. Only MAGs from this that passed the following parameters were used for annotation with BAKTA^32^, based on MIMAG (minimum information about a metagenome-assembled genome)^30^: completion >90% and contamination <10%. One MAG met the standard for a medium quality draft and was classified using standard nucleotide BLAST^29^ of the 23-16S sequence from the MAG, which matched *S. salivarius* 23S-16S sequence with 100% identity and 100% coverage. This genome was then examined for presence of the tryglysin biosynthetic operon and Rgg transcriptional regulator using protein BLAST^29^. This analysis was carried out on the Iowa State University Nova HPC and a personal computer.

### ITS and 16S amplicon sequencing and data processing

Genomic DNA was PCR amplified with primers sIDTP5_ITS1F and sIDTP7_ITS2R (CTACACGACGCTCTTCCGATCTCTTGGTCATTTAGAGGAAGTAA and CAGACGTGTGCTCTTCCGATCTGCTGCGTTCTTCATCGATGC, respectively – underlined regions represent linker sequences). Amplicons were generated using a two-stage PCR amplification protocol similar to that described previously^59^. The primers contained 5’ common sequence tags, underlined (sIDTP5 and sIDTP7) that match 3’ sequences present in IDT xGen™ Amplicon UDI primers (part numbers: 10009846, 10009851, 10009852, and 10009853). First stage PCR amplifications were performed in 10 microliter reactions in 96-well plates, using repliQa HiFi ToughMix (Quantabio). Genomic DNA input was 4 microliters per reaction. PCR conditions were 98 °C for 2 min, followed by 28 cycles of 98 °C for 10 min, 52 °C for 1 min and 68 °C for 1 min.

Subsequently, a second PCR amplification was performed in 10 µL reactions in 96-well plates using repliQa HiFi ToughMix. Each well received a separate primer pair containing unique dual indices (i.e., from the IDT xGen™ amplicon UDI primer sets). One microliter of PCR product from the first stage amplification was used as template for the 2nd stage, without cleanup. Two microliters of primer were used per reaction. Cycling conditions were 98 °C for 2 min, followed by 10 cycles of 98 °C for 10 min, 60 °C for 1 min and 68 °C for 1 min.

The PCR product was pooled prior to a 0.6X Ampure cleanup, followed by size selection on the Pippin Prep device (Sage Science) with 1.5% agarose gel. Size selected DNA fragments (300–750 bp) from the PippinPrep were sequenced with a 10% phiX spike-in on an Illumina Miniseq sequencer employing a mid-output flow cell (2 × 154 paired-end reads). Based on the results of the MiniSeq sequencing run, samples were re-pooled to balance output evenly among samples. The pooled products were cleaned as described above (i.e., Pippin Prep and Ampure cleanups) and sequenced on an Illumina NovaSeq6000 instrument using a 500-cycle SP flow cell with read lengths extended to 2 × 259 bases. Library preparation, pooling, and MiniSeq sequencing were performed at the Genomics and Microbiome Core Facility (GMCF) at Rush University. NovaSeq sequencing was performed at the DNA Services Facility at the Roy J. Carver Biotechnology Center at the University of Illinois at Urbana-Champaign.

For 16S sequencing, genomic DNA was prepared and sequencing using the same method as for ITS sequencing, with the following changes. 16S sequences were PCR amplified with primers sIDTP_515F and sIDTP7_806R (CTACACGACGCTCTTCCGATCTGTGCCAGCMGCCGCGGTAA and CAGACGTGTGCTCTTCCGATCTGGACTACHVGGGTWTCTAAT – underlined regions represent linker sequences), respectively. For 16S sequencing, only the 0.6X Ampure cleanup was performed after the second round of PCR amplification before sequencing on the Illumina Miniseq sequencer, no PippinPrep size selection was performed. All other steps were the same as performed for ITS sequencing. Resulting sequencing data were then imported into QIIME 2 v.2024.5^60^ as demultiplexed sequences. Phred qualities of sequences were examined before proceeding with QIIME2 analysis, using a custom script. Sequences were trimmed using the q2-cutadapt trim-paired plugin with according primers using cutadapt^61^ followed by processing in one of the two manners: quality filtering via the q-score using q2-quality-filter-q-score plugin and then using Deblur^62^ (via q2-deblur) or alternatively using DADA2^63^ (via q2-dada2) to perform clustering, chimera filtering, and abundance filtering. The resulting table output from Deblur or DADA2 was used to select a set sampling depth for analysis of samples, which was set the same for all samples within an individual experiment. Selection of appropriate sample depth was determined via examination of alpha rarefaction values for the selected sampling depth using q2-diversity-alpha-rarefaction plugin. Phylogenetic trees were generated for diversity analysis using q2-phylogeny align-to-tree-mafft-fasttree using MAFFT and fasttree2^64,65^, which was then used to examine alpha and beta diversity metrics in QIIME 2. Alpha diversity metrics generated included Faith’s Phylogenetic Diversity, Chao1 index, observed features, Simpson’s Dominance, and Shannon entropy^66–69^. Beta diversity metrics and Principle Coordinate Analysis (PCoA) were estimated using q2-diversity, and included Jaccard and Bray-Curtis distance^70^. Alpha diversity statistical significance was determined via a Kruskal-Wallis pairwise test with a Benjamini & Hochberg correction^50^, while beta diversity statistical significance was determined using PERMANOVA (adonis function)^51^. Statistical significance was determined if *q* < 0.05. To determine genus presence within samples, taxonomy was assigned to ASVs using the q2-feature-classifier^71^ classifier-sklearn naïve Bayes taxonomy classifier or q2-feature-classifier classify-consensus-blast as follows: for 16S results against the Greengenes 2022.10 full length sequences^72^; for ITS results against the QIIME UNITE database v.10.0 (released 2024-04-04^73^). The QIIME UNITE database classifier was trained using the Nova High Performance computing cluster (HPC) at Iowa State University, while the QIIME Greengenes classifier was trained on a personal computer. Resulting classification bar plots, alpha, and beta diversity analysis were visualized using the QIIME2 View online platform^60^. PCoA plot results generated via QIIME 2 were imported into R Studio using the qiime2R package v0.99.6 (Bisanz, JE. GitHub) and plotted using ggplot2^47^.

Before deposition of 16S and ITS sequence results to NCBI SRA, sequences were screened and filtered for potential human host sequences using Bowtie2^53^ on the Iowa State University Nova HPC with the GRCh37 human genome (NCBI RefSeq assembly: GCF_000001405.13). All raw sequence reads for experiments are deposited to the NCBI SRA under BioProject PRJNA1189147.

## Supplementary information

Supplementary Data 1

Supplementary Data 2

Supplementary Data 3

Supplementary Data 4

Supplementary Data 5

Supplementary Data 6

Supplementary Data 7

Supplementary Data 8

Supplementary Data 9

Supplementary Data 10

Supplementary Data 11

Supplementary Data 12

Supplementary Data 13

Supplementary Data 14

## Data Availability

All shotgun metagenomics data, ITS, and 16S data has been deposited to the NCBI Sequence Read Archive (SRA) under BioProject PRJNA1189147.

## Code availability

All codes and packages are available via their respective citations in *Methods*. The custom Linux shell script used to examine Phred score is available on GitHub (https://github.com/b-rued/Phred-check).
