# Supplementary material for: Impact of the RaS-RiPP tryglysin and culturing conditions on ex-vivo oral microbiomes
Source: NPJ Biofilms Microbiomes. 2025 Aug 4;11:152. doi: 10.1038/s41522-025-00794-8 (PMC12322106; doi:10.1038/s41522-025-00794-8)
Supplement: Supplementary file 1 — Supplementary Data 1 [file 41522_2025_794_MOESM1_ESM.pdf]

Supplementary Data 1

Impact of the RaS-RiPP tryglysin and culturing conditions on *ex-vivo* oral microbiomes.

Britta E. Rued[a#], Achal Dhariwal[b], Brett C. Covington[c], Mohammad R. Seyedsayamdost[c], Sophie A. Krivograd[a], Russell P. Pesavento[d], Michael J. Federle[e] and Fernanda C. Petersen[b#].

[a]Department of Veterinary Microbiology and Preventive Medicine. Iowa State University, Ames, Iowa, USA.

[b]Institute of Oral Biology. University of Oslo, Oslo, Norway.

[c]Department of Chemistry. Princeton University, Princeton, New Jersey, USA.

[d]College of Dentistry. Department of Oral Biology. University of Illinois - Chicago, Illinois, USA.

[e]Department of Pharmaceutical Sciences. University of Illinois - Chicago, Chicago, Illinois, USA

#Address correspondence to Britta E. Rued, brued@iastate.edu or Fernanda C. Petersen, f.c.petersen@odont.uio.no.

SUPPLEMENTAL FIGURES

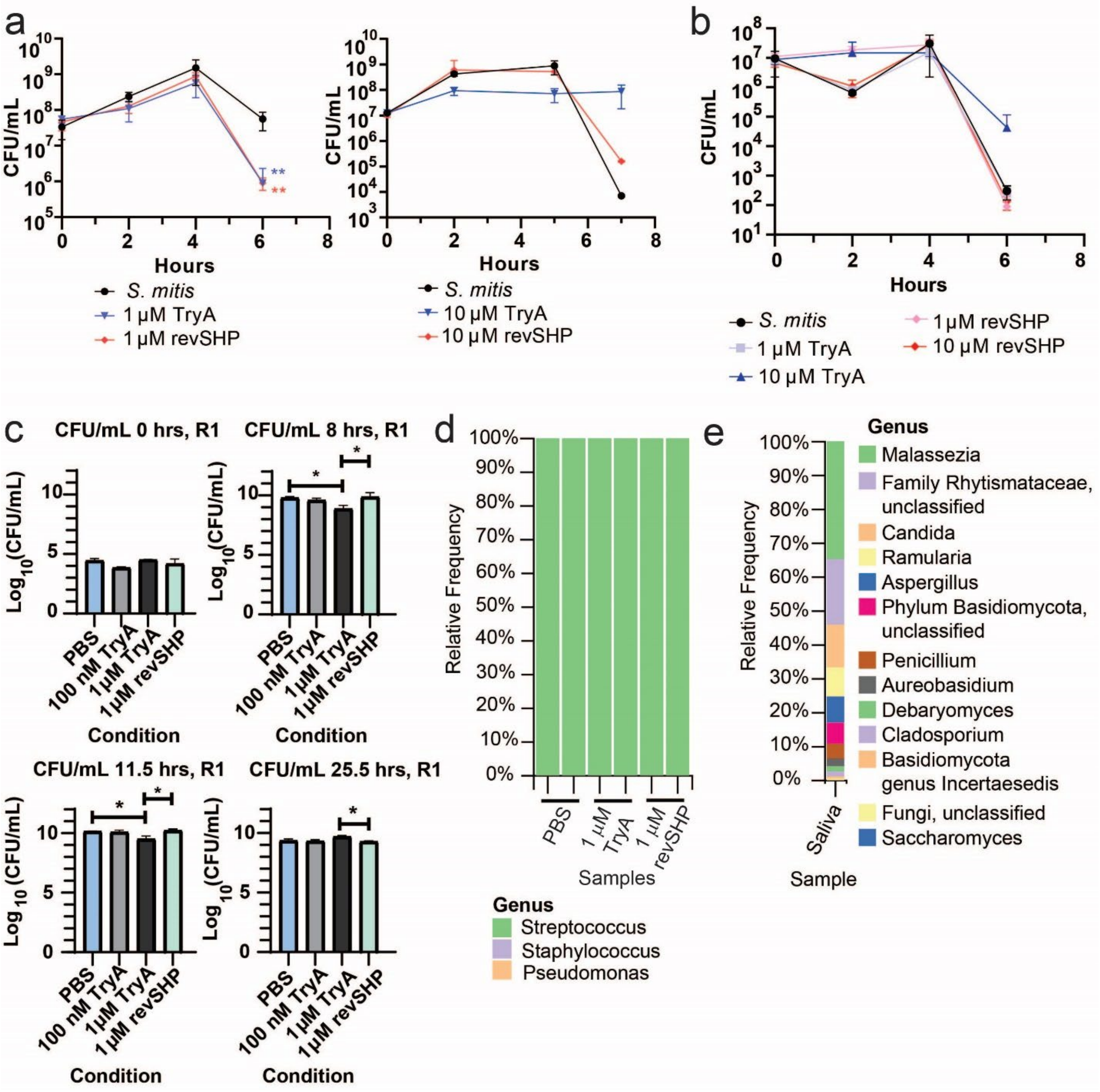

FIGURE S1: Additional data on initial salivary inoculum and examining Tryglysin A inhibition in SHI medium. Statistical significance was determined using a One-way ANOVA with Tukey's Multiple Comparisons Post-test; *, p-value < 0.05: **, p-value < 0.005. if values are not indicated comparisons were nonsignificant. A) CFU/mL of wild-type *S. mitis* from Norway laboratory exposed to increasing TryA, in SHI media.

Concentrations of TryA added are indicated below the graph. Statistical significance compared to the *S. mitis* control condition is indicated by symbols colored corresponding to the panel legend. This experiment was performed twice: once with 1 μM TryA, once with 10 μM TryA. Graphs for both experiments are shown. B) CFU/mL of wild-type *S. mitis* from Chicago laboratory exposed to increasing TryA in SHI. Concentrations of Tryglysin A are indicated in the legend below the graph and correspond to the respective symbol types. No statistical significance was observed. This experiment was performed three times with similar results. C) CFU/mL bar graphs of salivary inoculum in CDM medium exposed to increasing TryA, PBS, or revSHP. This experiment was performed twice. Data correlates to graph shown in Fig. 1C. D) 16S rRNA sequencing results of samples grown in CDM for 24 hours, with PBS, 1 μM Tryglysin A, or 1 μM revSHP (*S. mutans* reverse SHP, *Methods*). E) Fungal genera detected in human pooled saliva isolates as determined by ITS sequencing from Norway isolation. Relative abundance, genera are indicated by color as detailed in the legend beside the graph. The Norwegian pooled saliva was sequenced once.

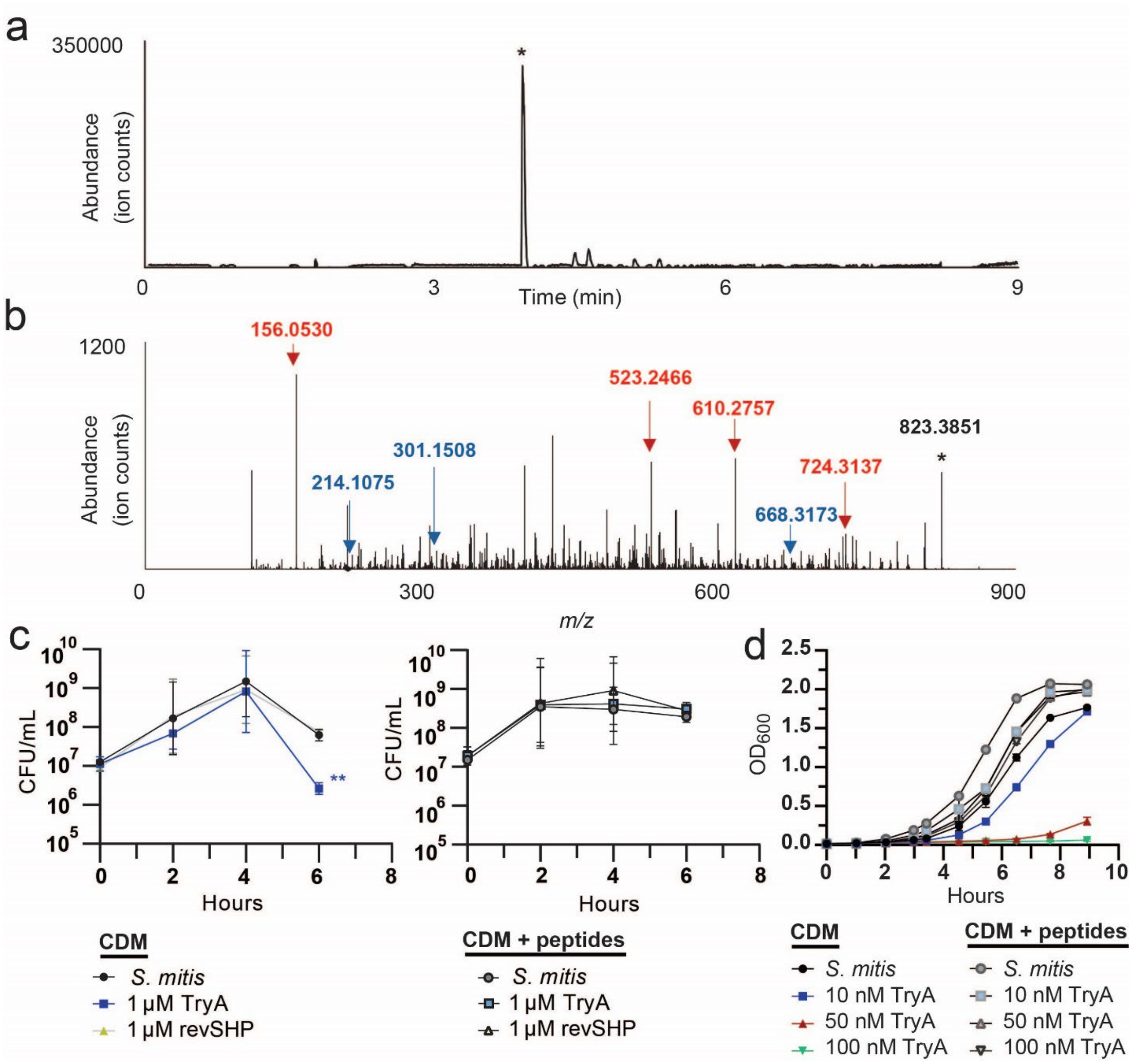

FIGURE S2: Data for confirmation of purified Tryglysin A and experiments examining the effect of excess peptides on Tryglysin A activity. A) HPLC trace, blank subtracted base peak chromatogram for purified Tryglysin A. The peak corresponding to Tryglysin A is marked with "*". B) MS/MS spectra for purified Tryglysin A. The parent ion (*m/z*: 823.3851) is marked with "*". Important fragment ions are marked by corresponding *m/z* values and indicated with arrows. Blue arrows indicate b-ions, and red arrows indicate y-ions. The fragmentation pattern and the intensity of observed fragments ions are

entirely consistent with the previously reported structure of tryglysin A. C) CFU/mL of wild-type *S. mitis* exposed to increasing TryA, in CDM or CDM supplemented with peptides equivalent to SHI medium. Statistical significance compared to control condition was determined using a One-way ANOVA with Tukey's Multiple Comparisons Post-test; ** p-value < 0.005; if values are not indicated they are non-significant. Statistical significance is color-coded to match the corresponding figure legend. This experiment was repeated three times with similar results. D) Growth curve of wild-type *S. mitis* exposed to increasing TryA, in CDM or CDM supplemented with peptides equivalent to SHI medium. This experiment was repeated three times with similar results.

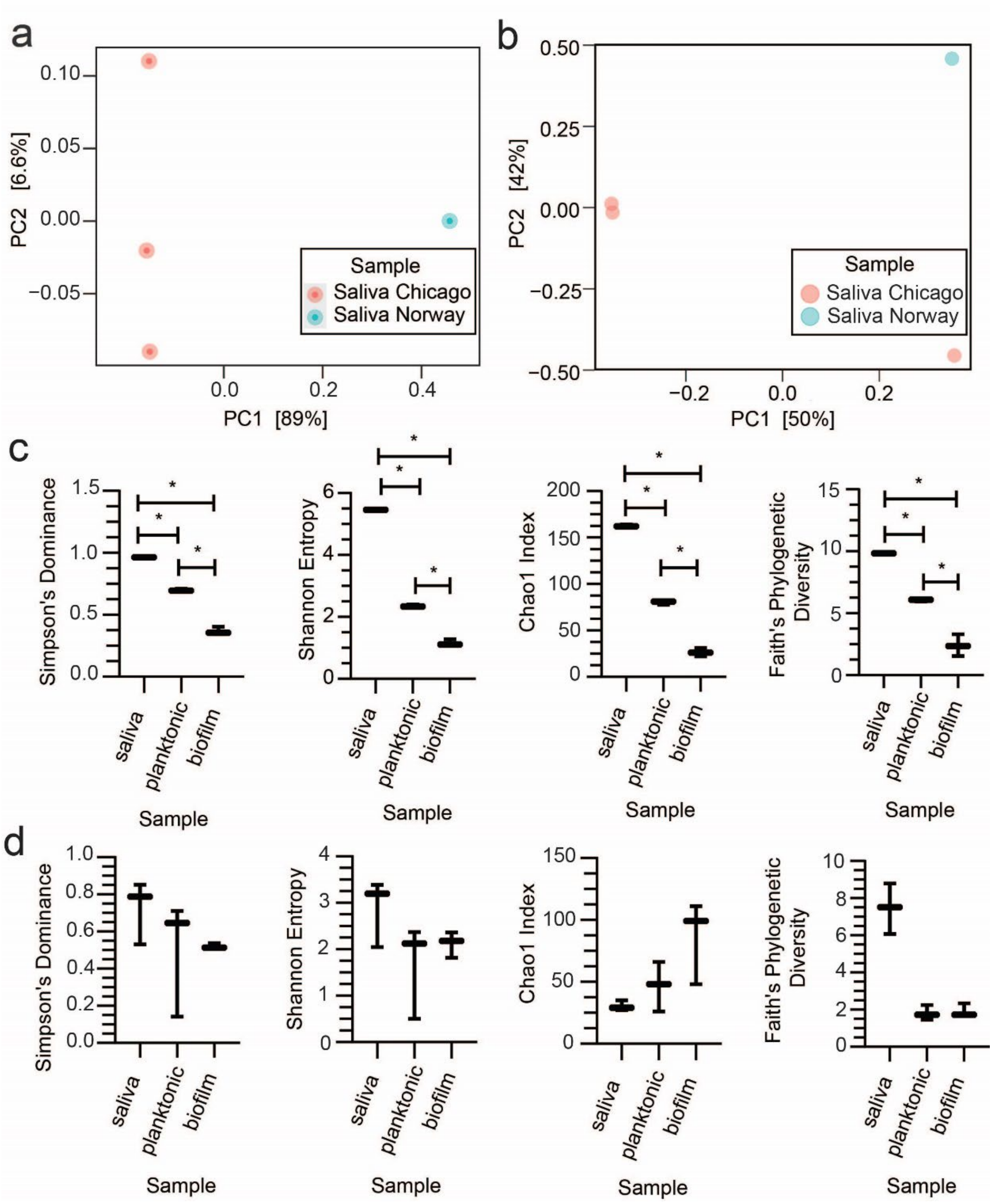

FIGURE S3: PCoA plots comparing pooled saliva collections from Norway and Chicago, and alpha diversity metrics for 16S and ITS sequencing from *ex-vivo* oral samples cultured with 5% CO_2. For pooled saliva collections, the Norway pooled saliva was sequenced once, whereas the Chicago pooled saliva was sequenced in triplicate, with all samples originating from DNA extracted from the same salivary pool. For panels C and

D, statistical significance between conditions was determined using a pairwise Kruskal-Wallis test with a p-value adjustment using a Benjamini & Hochberg correction. * indicates q-value < 0.05. If no symbols are present, this indicates comparisons were non-significant. A) Jaccard PCoA of Chicago and Norway pooled saliva from shotgun metagenomics. B) Bray-Curtis PCoA of Chicago and Norway pooled saliva from ITS sequencing. C) Alpha diversity metrics for 16S sequencing results shown in Fig. 3B. D) Alpha diversity metrics for the ITS sequencing shown in Fig. 3C-D.

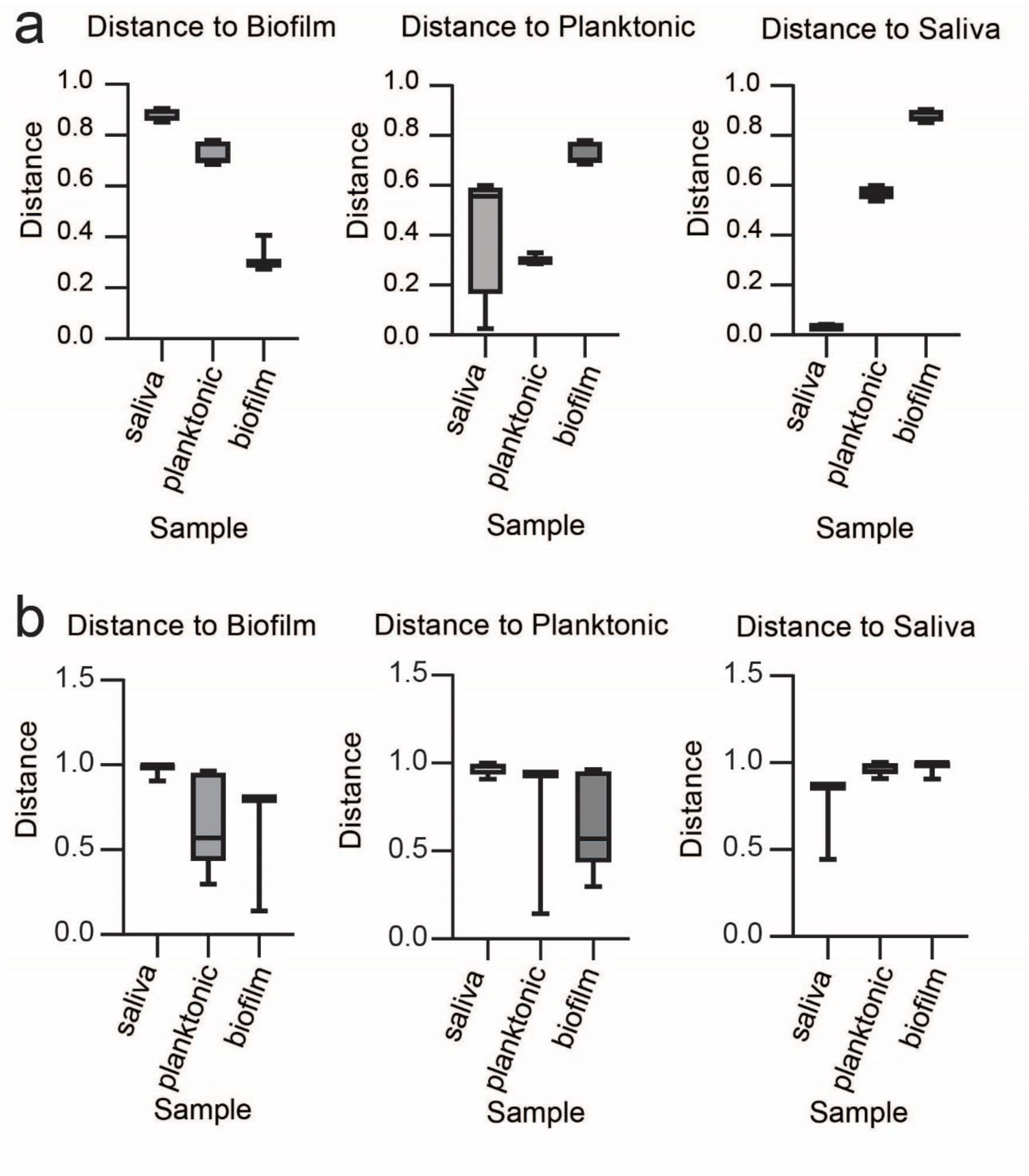

FIGURE S4: Beta diversity metrics for 16S and ITS sequencing from *ex-vivo* oral samples cultured with 5% CO_2. Statistical significance between conditions was determined using PERMANOVA (adonis function). Pairwise comparisons are indicated between samples by brackets. If no brackets are present, this indicates comparisons were non-significant. A) Beta diversity metrics determined via Jaccard distance for 16S sequencing results

shown in Fig. 3B. B) Beta diversity metrics determined via Bray-Curtis distance for ITS sequencing results shown in Fig. 3C-3D.

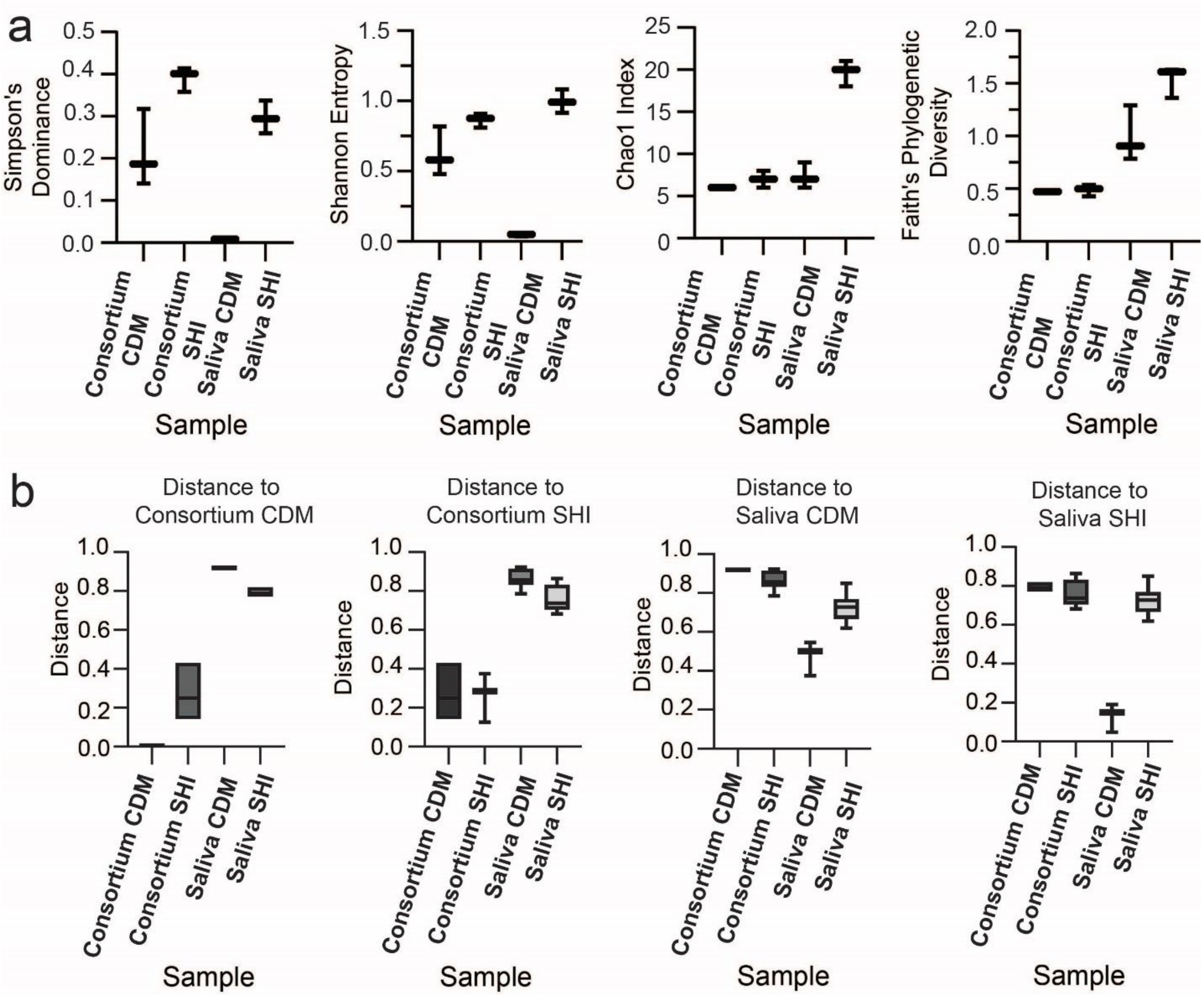

FIGURE S5: Additional data examining the impact of CDM vs. SHI media on bacterial population for culturing *ex-vivo* oral microbiomes. Samples were grown in CDM or SHI media for 24 hours with 5% CO_2, in biological triplicate. Note the differences in scales on the y-axis for graphs. Data correlates with 16S sequencing results shown in Fig. 4C-4D. A) Alpha diversity metrics with samples split by inoculum source and media. Statistical significance between conditions was determined using a pairwise Kruskal-Wallis test with a p-value adjustment using a Benjamini & Hochberg correction. If no symbols are present, this indicates comparisons were non-significant. B) Beta diversity metrics using Jaccard

distance with samples split by inoculum source and media. Statistical significance between conditions was determined using pairwise PERMANOVA (adonis function). Pairwise comparisons are indicated between samples by brackets. If no brackets are present, this indicates comparisons were non-significant.

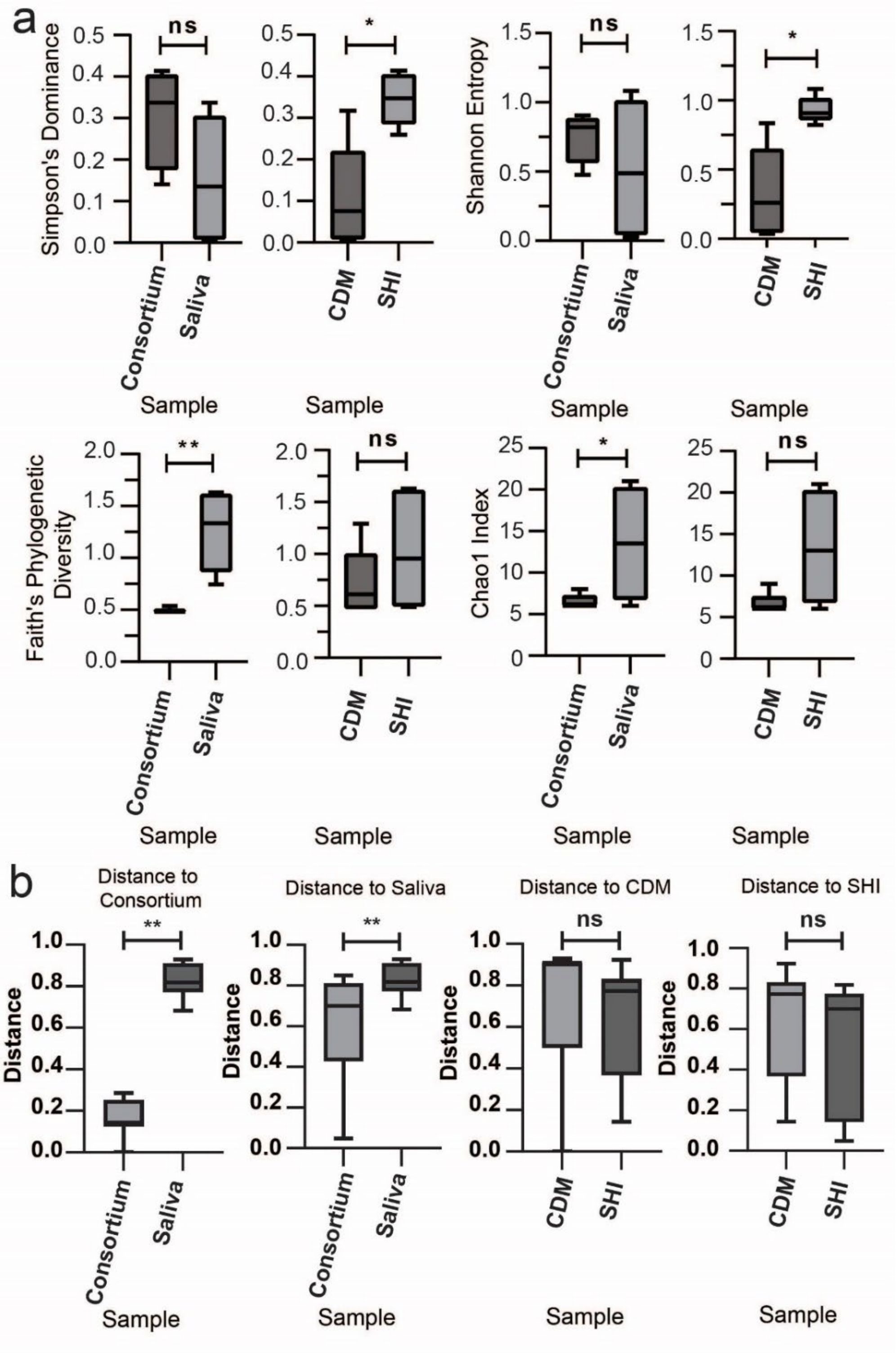

FIGURE S6: Comparison of additional alpha and beta diversity metrics for 16S data shown in Fig. 4C-4D. Shown are two other comparisons: consortia vs. saliva (inoculum

source), or CDM vs. SHI media (medium condition). For all graphs, note the differences in scales on the y-axis. A) Alpha diversity metrics for additional comparisons. Statistical significance between conditions was determined using a pairwise Kruskal-Wallis test with a p-value adjustment using a Benjamini & Hochberg correction. *, q-value < 0.05; **, q-value < 0.005; ns, nonsignificant. B) Beta diversity metrics using Jaccard distance for additional comparison. Statistical significance between conditions was determined using pairwise PERMANOVA (adonis function). **, q-value < 0.005; ns, nonsignificant.

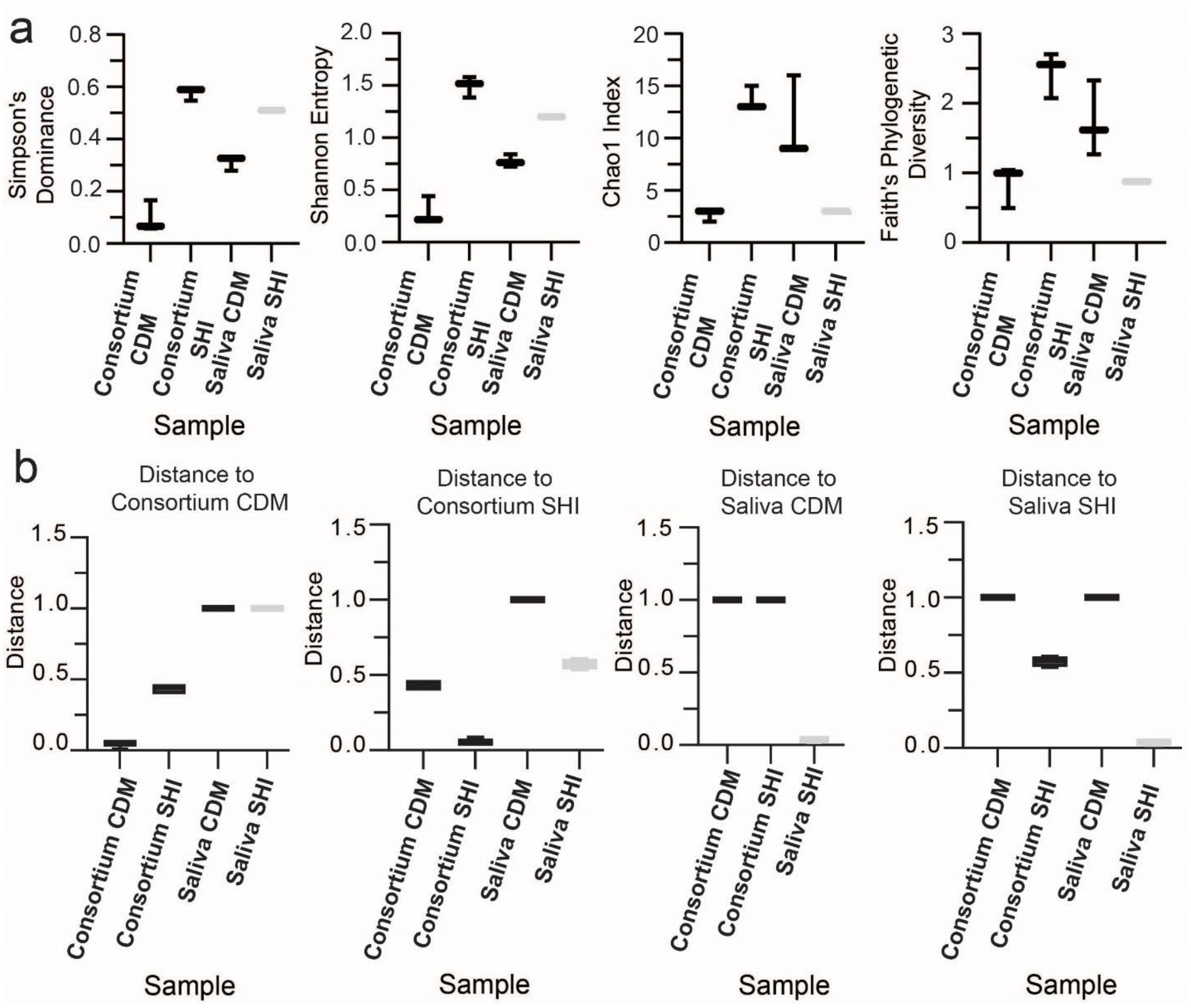

FIGURE S7: Additional data examining the impact of CDM vs. SHI media on fungal population for culturing *ex-vivo* oral microbiomes. Samples were grown in CDM or SHI media for 24 hours with 5% CO_2, in biological triplicate. Note the differences in scales on the y-axis for graphs. Data correlates with ITS sequencing results shown in Fig. 5A-5B. A) Alpha diversity metrics with samples split by inoculum source and media. Statistical significance between conditions was determined using a pairwise Kruskal-Wallis test with a p-value adjustment using a Benjamini & Hochberg correction. If no symbols are present, this indicates comparisons were non-significant. B) Beta diversity metrics using Bray-

Curtis distance with samples split by inoculum source and media. Statistical significance between conditions was determined using PERMANOVA (adonis function). Pairwise comparisons are indicated between samples by brackets. If no brackets are present, this indicates comparisons were non-significant.

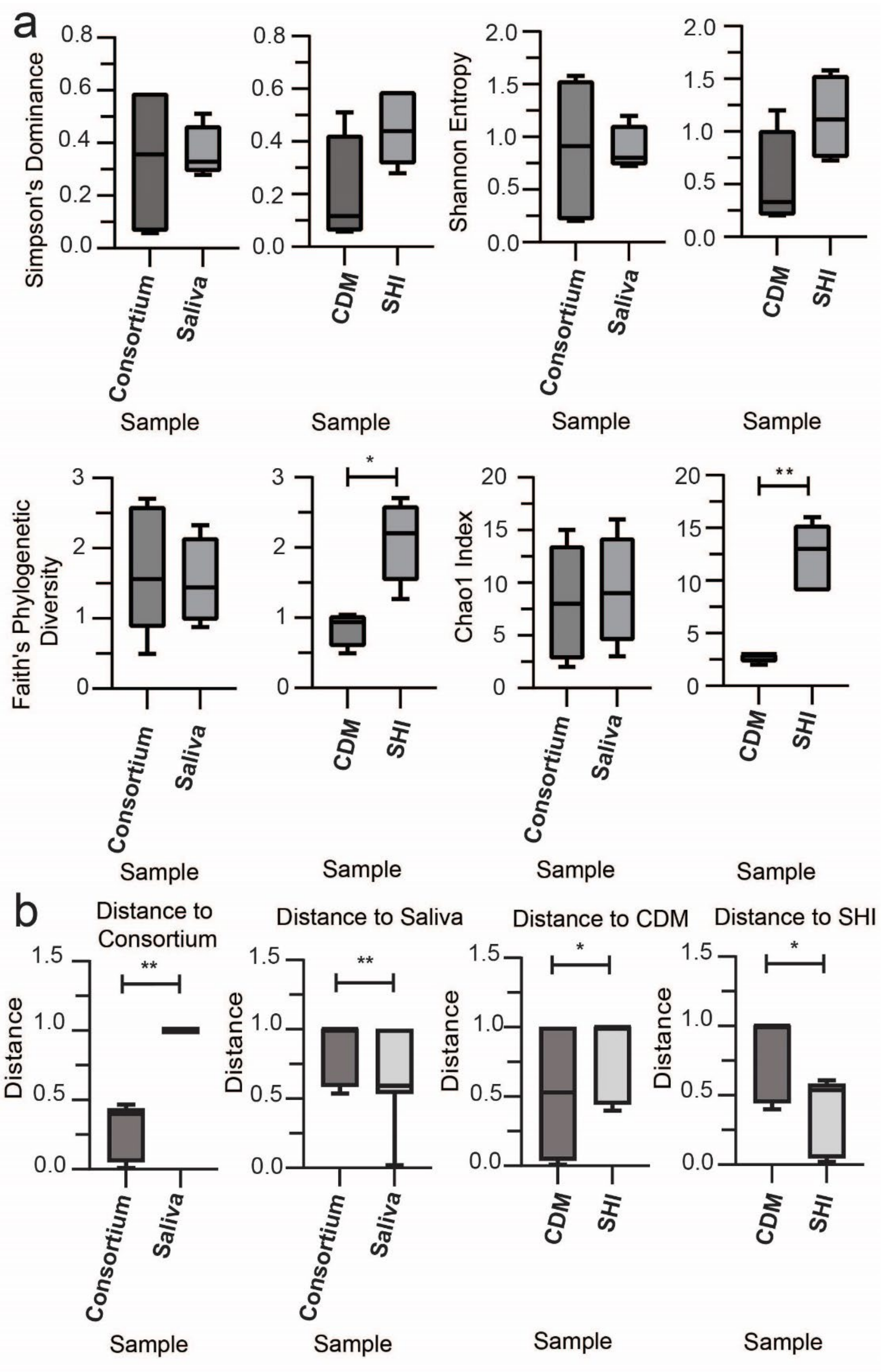

FIGURE S8: Comparison of additional alpha and beta diversity metrics for ITS data shown in Figure 5A-5B. Shown are two other comparisons: consortia vs. saliva, or CDM

vs. SHI media. For all graphs, note the differences in scales on the y-axis. A) Alpha diversity metrics for additional comparisons. Statistical significance between conditions was determined using a pairwise Kruskal-Wallis test with a p-value adjustment using a Benjamini & Hochberg correction. *, q-value < 0.05; **, q-value < 0.005; no symbols present, nonsignificant. B) Beta diversity metrics using Bray-Curtis distance for additional comparisons. Statistical significance between conditions was determined using pairwise PERMANOVA (adonis function). *, q-value < 0.05; no symbols present, nonsignificant.

TABLE S1. Observed b- and y- ions from Tryglysin A MS/MS analysis.[a]

Ion	Calculated *m/z*	Observed *m/z*	Δppm	Unmodified Sequence
y_6^{+1}	724.3183	724.3137	6	NSWGKH
y_5^{+1}	610.2754	610.2757	0.5	SWGKH
y_4^{+1}	523.2434	523.2466	6.1	WGKH
y_3^{+1}	339.1786	---	---	GKH
y_2^{+1}	282.1572	---	---	KH
y_1^{+1}	156.0767	156.053	151.8	H
b_6^{+1}	668.3173	668.3102	10.6	VNSWGK
b_5^{+1}	542.2368	---	---	VNSWG
b_4^{+1}	485.2154	---	---	VNSW
b_3^{+1}	301.1507	301.1508	0.3	VNS
b_2^{+1}	214.1186	214.1075	51.8	SVN
b_1^{+1}	100.0757	---	---	V
MH^{+1}	823.3867	823.3851	1.9	VNSWGKH

[a]Calculated *m/z* values include the loss of 2 H atoms from tryptophan and 2 H atoms from lysine to account for the lysine-tryptophan crosslink in Tryglysin A.

TABLE S2. Bacterial strains used in this study.[a]

Bacterial strains			
**Strain**	**Description**	**Antibiotic Resistance**	**Reference**[b]
S. mitis	*Streptococcus mitis* CCUG 31611 wild-type isolate, also known as NCTC 12261	None	Carlsson 1968. *Odontol Revy.*
S. salivarius	*Streptococcus salivarius* 101-1	None	Federle lab, unpublished oral isolate
S. ferus	*Streptococcus ferus* DSM20646	None	Bushin *et al.,* 2018. *JACS.*

[a]Strains were used as described in *Methods*.
[b]See supplementary reference list.

SUPPLEMENTARY REFERENCES

1. Carlsson J. A numerical taxonomic study of human oral streptococci. *Odontol Revy*. 1968;19(2):137-60. PMID: 4387086.
2. Bushin LB, Clark KA, Pelczer I, Seyedsayamdost MR. 2018. Charting an Unexplored Streptococcal Biosynthetic Landscape Reveals a Unique Peptide Cyclization Motif. J Am Chem Soc 140:17674–17684.

ADDITIONAL SUPPLEMENTARY DATA LEGENDS

SUPPLEMENTARY DATA 2: Relative abundances from MetaPhlAn3 analysis of bacterial species detected during culturing of Norway saliva in CDM. Note that 1 = 100% relative abundance, Sample codes: MB-BR1_S442, PBS R1; MB-BR2_S443, PBS R2; MB-BR3_S444, 1 μM TryA R1; MB-BR4_S445, 1 μM TryA R2; MB-BR5_S446, 1 μM revSHP R1; MB-BR6_S447, 1 μM revSHP R2. *Note that 1 = 100% relative abundance

SUPPLEMENTARY DATA 3: DESeq2 statistical analysis of MetaPhlAn3 estimated count abundances from culturing of Norway saliva in CDM. Legend key: baseMean, Average of all normalized count values; log2FoldChange, Effect size estimate of change between groups; lfcSE, Standard error estimate for log2 fold change estimate; stat, The value of the test statistic for the species; pvalue, P-value of the test for the species; padj, Adjusted P-value of the test for the species, using Benjamini and Hochberg method.

SUPPLEMENTARY DATA 4: Alpha diversity metrics of MetaPhlAn3 estimated count abundances from culturing of Norway saliva in CDM.

SUPPLEMENTARY DATA 5: Metagenome assembled genome binning statistics and taxonomic assignment per sample if performed. Sample codes: MB-BR1_S442, PBS R1; MB-BR2_S443, PBS R2; MB-BR3_S444, 1 μM TryA R1; MB-BR4_S445, 1 μM TryA

R2; MB-BR5_S446, 1 µM revSHP R1; MB-BR6_S447, 1 µM revSHP R2. NA: Taxonomic and genome assignment was only performed on metagenome assemblies with completeness of <90% and contamination >10%.

SUPPLEMENTARY DATA 6: Relative abundances from MetaPhlAn3 analysis of bacterial species in uncultured pooled Norway saliva. Sample code: GMCF_2339b_01_S1_S264, Initial Saliva. *Note that 1 = 100% relative abundance

SUPPLEMENTARY DATA 7: Relative abundance from MetaPhlAn3 analysis of Chicago saliva bacterial species detected. Sample codes: BR-A35_S160, Planktonic SHI media R1; BR-A36_S161, Planktonic SHI media R2; BR-A37_S162, Planktonic SHI media R3; BR-A41_S163, Biofilm SHI media R1; BR-A42_S164, Biofilm SHI media R2; BR-A43_S165, Biofilm SHI media R3; BR-SA1_S157, Initial Saliva R1; BR-SA2_S158, Initial Saliva R2; BR-SA3_S159, Initial Saliva R3. *Note that 1 = 100% relative abundance

SUPPLEMENTARY DATA 8: Fungal genera detected and summary of ASV counts from QIIME2 analysis of ITS sequencing data of pooled Norway saliva sample.

SUPPLEMENTARY DATA 9: Fungal genera detected and summary of ASV counts from QIIME2 analysis of ITS sequencing data from propagation of Chicago saliva with 5% CO_2.

SUPPLEMENTARY DATA 10: Comparisons of alpha and beta group diversity metrics from QIIME2 analysis for ITS Norway and ITS Chicago initial saliva collection.

SUPPLEMENTARY DATA 11: Bacterial genera detected and summary of ASV counts from QIIME2 analysis of 16S sequencing data from propagation of Chicago saliva with 5% CO_2.

SUPPLEMENTARY DATA 12: Comparison of phyla and genera detected in Edlund *et al.,* 2013 study versus our study's planktonic and biofilm models. *Note that Lactobacillus was split into several different genera as of 2020. See Zheng *et al*., 2020, *Internat. J. of Systemic and Evolutionary Microbiology.* **TM7 was renamed Saccharibacteria. See Bor *et al.,* 2019. *J Dent. Res.* ND indicates that classification has changed, is undetermined, or that genera has been identified since 2013 and samples could have been present. 0 indicates no samples were detected. Red indicates that the genera was detected in only one sample of three replicates.

SUPPLEMENTARY DATA 13: Bacterial genera detected and summary of ASV counts from QIIME2 analysis of 16S sequencing data from propagation of Chicago saliva or propagated consortia with 5% CO_2. *Due to the observation of this genera in only one replicate out of three, this was not considered a true result.

SUPPLEMENTARY DATA 14: Fungal genera detected and summary of ASV counts from QIIME2 analysis of ITS sequencing data from propagation of Chicago saliva or propagated consortia with 5% CO_2.
